# A comparative analysis of heart microRNAs in vertebrates brings novel insights into the evolution of genetic regulatory networks

**DOI:** 10.1186/s12864-021-07441-4

**Published:** 2021-03-04

**Authors:** Pedro G. Nachtigall, Luiz A. Bovolenta, James G. Patton, Bastian Fromm, Ney Lemke, Danillo Pinhal

**Affiliations:** 1 Laboratório Especial de Toxinologia Aplicada (LETA), CeTICS, Instituto Butantan, São Paulo, Brazil; 2 Department of Chemical and Biological Sciences, Institute of Biosciences of Botucatu, São Paulo State University (UNESP), Botucatu, Brazil; 3 Department of Biophysics and Pharmacology, Institute of Biosciences of Botucatu, São Paulo State University (UNESP), Botucatu, Brazil; 4 Department of Biological Sciences, Vanderbilt University, Nashville, USA; 5 Department of Molecular Biosciences, The Wenner-Gren Institute (MBW), Stockholm University, Stockholm, Sweden

**Keywords:** Small RNA, Non-coding RNA, Functional genomics, Comparative genomics, Cardiac miRNAs, Genetic regulatory network

## Abstract

**Background:**

During vertebrate evolution, the heart has undergone remarkable changes that lead to morphophysiological differences in the fully formed heart of these species, such as chamber septation, heart rate frequency, blood pressure, and cardiac output volume. Despite these differences, the heart developmental process is guided by a core gene set conserved across vertebrates. Nonetheless, the regulatory mechanisms controlling the expression of genes involved in heart development and maintenance are largely uncharted. MicroRNAs (miRNAs) have been described as important regulatory elements in several biological processes, including heart biology. These small RNA molecules are broadly conserved in sequence and genomic context in metazoans. Mutations may occur in miRNAs and/or genes that contribute to the establishment of distinct repertoires of miRNA-target interactions, thereby favoring the differential control of gene expression and, consequently, the origin of novel phenotypes. In fact, several studies showed that miRNAs are integrated into genetic regulatory networks (GRNs) governing specific developmental programs and diseases. However, studies integrating miRNAs in vertebrate heart GRNs under an evolutionary perspective are still scarce.

**Results:**

We comprehensively examined and compared the heart miRNome of 20 species representatives of the five major vertebrate groups. We found 54 miRNA families with conserved expression and a variable number of miRNA families with group-specific expression in fishes, amphibians, reptiles, birds, and mammals. We also detected that conserved miRNAs present higher expression levels and a higher number of targets, whereas the group-specific miRNAs present lower expression levels and few targets.

**Conclusions:**

Both the conserved and group-specific miRNAs can be considered modulators orchestrating the core and peripheral genes of heart GRNs of vertebrates, which can be related to the morphophysiological differences and similarities existing in the heart of distinct vertebrate groups. We propose a hypothesis to explain evolutionary differences in the putative functional roles of miRNAs in the heart GRNs analyzed. Furthermore, we present new insights into the molecular mechanisms that could be helping modulate the diversity of morphophysiology in the heart organ of vertebrate species.

**Supplementary Information:**

The online version contains supplementary material available at (10.1186/s12864-021-07441-4).

## Background

In vertebrates, the heart is responsible for the continuous blood flow, which is crucial for the life of these organisms. This organ is the first to form and function in the developing embryo [1]. Noteworthy, the heart, and the cardiovascular system as a whole, have undergone many morphophysiological changes during vertebrate evolution (reviewed by [2]). In fishes, the heart consists of two chambers, one atrium, and one ventricle. Amphibians present a three-chambered heart (i.e., two atrium and one ventricle). The heart of Sauria, which can be split into Lepidosauria clade, represented by lizards and snakes, that presents a similar heart morphology to the amphibian representatives with partial divisions of the ventricle, and Archosauria, represented by turtles, crocodilians, and birds, which turtles present a similar morphology to Lepidosauria whereas crocodilians and birds present full septation of the ventricle, similar to what is found in mammals [3, 4]. Although birds and lizards are part of the monophyletic clade of Sauria, we will refer to lizards as “reptiles”, due to the differences in the heart morphology and the control of body temperature characteristic. Mammals evolved a four-chambered heart with a fully septated ventricle, endothermy, and complete division between the pulmonary and systemic blood circulation, which is shared with the bird representatives. Interestingly, the endothermy and a four-chambered fully septated heart in mammals and birds are a good example of convergent evolution. The evolution of such morphological traits was accompanied by an increase in systemic blood pressure, heart rate and cardiac output volume, which is considered a pivotal biological trait to sustain the inherent higher metabolism required by endothermy [5, 6].

Although morphological differences are inherent to the adult heart, it is known that the heart developmental process is highly similar among vertebrates, suggesting conserved mechanisms regarding the building plan architecture of the heart. At the molecular level, the core program for heart development is driven by a complex and precise process involving thousands of genes working into genetic regulatory networks (GRNs) that coordinate the cardiogenesis [1, 7]. The heart GRNs are based on logic circuits with each part subjected to a fine-tuned expression culminating into the final morphophysiology of the organ. The assembly of GRNs is important for identification of particular genes involved in specific phenotypes and diseases, and to improve our understanding on evolution of complex traits [8]. The main concept of the vertebrate phenotypic evolution is related to the refinement of the expression level of developmental regulators [9]. For instance, the evolution of ventricular septation in mammals and birds was shown to be related with a fine-tuned expression of the transcriptional factor TBX5 [10]. However, the molecular mechanisms controlling the refinement in the expression of TBX5 and other important genes have yet to be fully uncovered. In fact, diverse interactions and regulatory mechanisms acting in the heart GRNs responsible for heart species-specific singularities remain unclear. Particularly, little is known about the role played by non-coding RNAs in shaping the heart distinctive morphology among species, both at the onset of heart formation and later in the adult heart.

MicroRNAs (miRNAs) are a large class of small non-coding RNAs acting as regulatory elements of gene expression in metazoan, plant, and viruses [11]. In general, these small molecules affect the final protein output through inhibition of translation and/or mRNA degradation by binding at the 3’UTR of their mRNA target [12, 13]. Target prediction analyses have shown that miRNA-mRNA interactions are conserved and the vast majority of mRNAs are under the regulation of one or multiple miRNAs [14]. These inferred interactions suggest that miRNAs are actively influencing multiple developmental processes and diseases. Indeed, miRNAs were shown to play key roles in heart development [15], and changes in miRNAs expression were related to heart abnormalities that lead to diseases and death [16, 17]. However, only a small fraction of miRNAs expressed in the heart of vertebrates have been deeply examined, implying that functional roles of miRNAs and bona-fide miRNA-target interactions in heart GRNs are still largely unknown.

Many miRNAs are broadly conserved in vertebrates [18], whereas several miRNAs are group-specific (i.e., specific to a single species or group of closely related species) [19–26]. This indicates that miRNAs can be actively participating in specific regulatory pathways associated with phenotypic differences observed among species, and that miRNAs are related to the establishment of tissues and organs morphophysiology [21, 27, 28]. In fact, several studies showed that knocking down the broadly conserved miRNA families leads to abnormal phenotypes (reviewed by [18]). Moreover, the disruption of a single miRNA-target interaction is sufficient to result in specific phenotypic abnormalities [29]. However, this affected interaction may lead to disruption of all other miRNA-target interactions, which can also be acting at any level to modulate the specific phenotypic abnormality observed [29]. All these data indicate that the whole set of miRNAs are important modulators across numerous GRNs governing the design of distinct phenotypes, including the GRNs responsible for the observed heart shape in the vertebrate species.

In order to understand the roles played by miRNAs in the evolution of heart GRNs of vertebrates, we used publicly available data from 17 vertebrate species and expanded the set of species analyzed by sequencing miRNAs from the heart of Nile tilapia, *Xenopus laevis*, and one lizard species. In this sense, we were able to comprehensively characterize and compare the heart miRNome of 20 vertebrate species, being nine mammals (i.e., one monotreme, one marsupial, and seven eutherians), two birds, one reptile, two amphibians, and six fishes. Our study sheds light on the evolutionary aspects of conserved and group-specific miRNAs acting on core and peripheral genes of the heart GRN that could be shaping the distinct heart phenotype of vertebrates.

## Results

### Heart miRNA expression, family characterization and comparative analysis

**Fig. 1:**
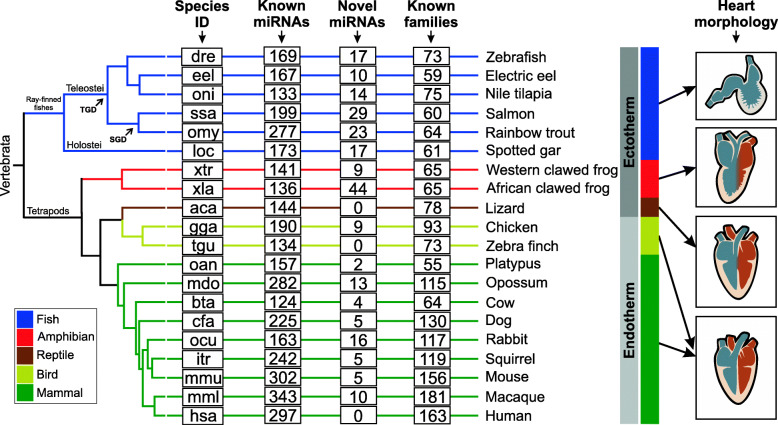
Heart miRNAs in vertebrates. Species ID is indicated at left. Known miRNAs are miRNAs with orthologs identified based on sequence similarity with miRNAs annotated in miRBase and MirGeneDB. Novel miRNAs are putative miRNAs identified in each species by our pipeline. Known families are based on miRBase and MirGeneDB annotations. Heart Morphology is a simplified representation of heart for each group of vertebrates (fishes: two-chambered heart and ectothermy; amphibians: three-chambered heart and ectothermy; reptiles: representative of the Lepidossauria clade presenting a three-chambered heart with partial division at the ventricle and ectothermy; birds: representatives of the Archosauria clade with four-chambered heart and endothermy; mammals: four-chambered heart and endothermy). TGD is Teleost-specific Genome Duplication. SGD is Salmonid-specific Genome Duplication. The phylogenetic tree is a handmade tree derived by merging tree available at TimeTree resource [30] and trees published by [31–33]

The assessment of the heart miRNome of 20 vertebrate species allowed for the identification of 153 to 534 miRNAs loci, depending on the species considered. From this total, 149 to 511 referred to known miRNAs, whereas 2 to 44 referred to putative novel miRNAs (Fig. 1; the results are summarized in Table S1 in Additional file 1 and detailed for each species in Additional file 2). The majority of miRNAs could be assigned to known families, whereas a few were not assigned to any family due to lack of sequence similarity. The identification of putative novel miRNAs not previously reported or annotated may reflect our exhaustive search on raw datasets and the differences in the distinct workflows applied in the present study and previous reports; however, it may also represent artifacts detected by our miRNA identification pipeline.

**Fig. 2:**
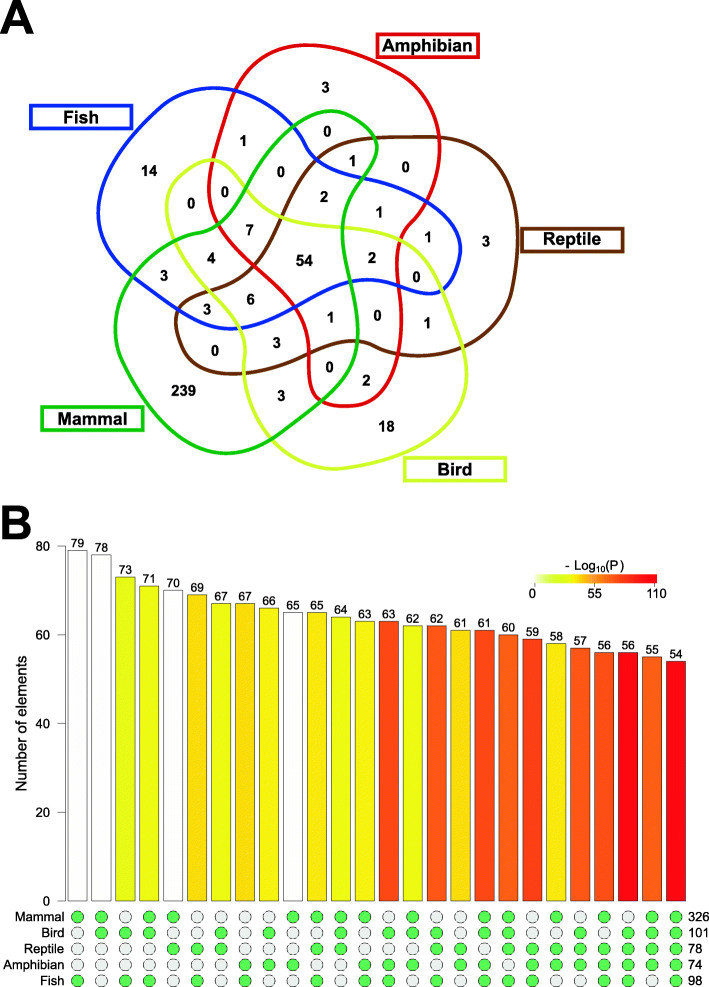
Intersections of vertebrate heart miRNA expression profile. (**a**) Venn diagram showing the intersections of vertebrate heart miRNA families. (**b**) Fisher’s exact test results for all intersections (p < 0.005 were considered statistically significant). The numbers at the right bottom indicate the number of miRNA families in the groups indicated at the left bottom. The numbers at the top of the bars indicate the number of miRNA families intersecting between the groups included for the statistical tests as stated by the green points at the bottom

Based on precursor sequence similarity, we assigned the miRNAs identified to 375 families (see Additional files 1 and 3 for further details), being 54 of them expressed into all five vertebrate groups (Fig. 2a; Table S2 in Additional file 1; referred to as conserved miRNAs). On the other hand, we detected a group-specific expression for 14, 3, 3, 18, and 239 miRNA families in fishes, amphibians, reptiles, birds, and mammals, respectively. Most of the intersections detected in this analysis were statistically significant when compared to the random expectation (*p*-value lower than 0.005; Fig. 2b; Table S3 in Additional file 1), indicating that conserved and group-specific miRNAs can be integrated into regulatory pathways driving the heart morphophysiology observed in vertebrates.

**Fig. 3:**
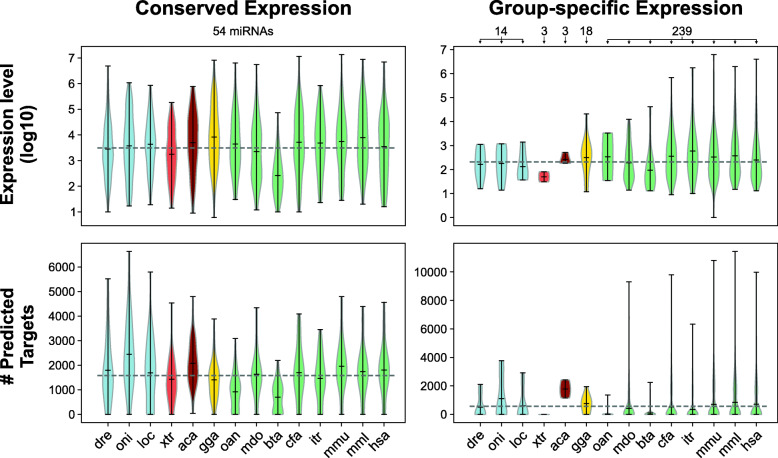
Expression level and the number of predicted targets of miRNAs with conserved and group-specific expression in the heart of vertebrates. The number of conserved and group-specific miRNAs analyzed in each species is indicated at the top of the plots. Violin plots of the expression level (top) and the number of predicted targets (bottom)

Tracing the birth age of miRNAs expressed in the heart of vertebrates revealed that conserved miRNA families have representatives that can be traced back to 400-690 Million Years Ago (MYA). Conversely, group-specific families stand for younger miRNA families (Table S2 in Additional file 1). We compared the expression level and number of predicted target genes for both the conserved and group-specific miRNA families (Fig. 3). Conserved families potentially presents an elevated number of putative targets (Wilcoxon rank-sum test W: 208, *p*-value = 1.555e-05), and higher expression levels (Wilcoxon rank-sum test W: 219, *p*-value = 3.868e-07), when compared do group-specific miRNAs. In this sense, our analysis suggests that conserved miRNAs, which present high expression and target several genes, may be acting on several processes in the heart GRN, whereas the group-specific miRNAs, which present lower expression and target few genes, may be fine-tuning specific processes.

### Predictions of microRNAs relevant to the control of the heart GRN

Our pipeline to identify miRNA-target interactions were designed to integrate both predictions of TargetScan and miRanda, followed by filtering genes not expressed in the heart. We also performed a comprehensive search in miRTarBase and scientific literature for validated interactions. We were able to generate a unique heart GRN for each vertebrate group analyzed. Results from all predicted and validated interactions identified along with the centrality analysis were organized in the Supplementary Tables S1–S12 in Additional file 4.

**Fig. 4:**
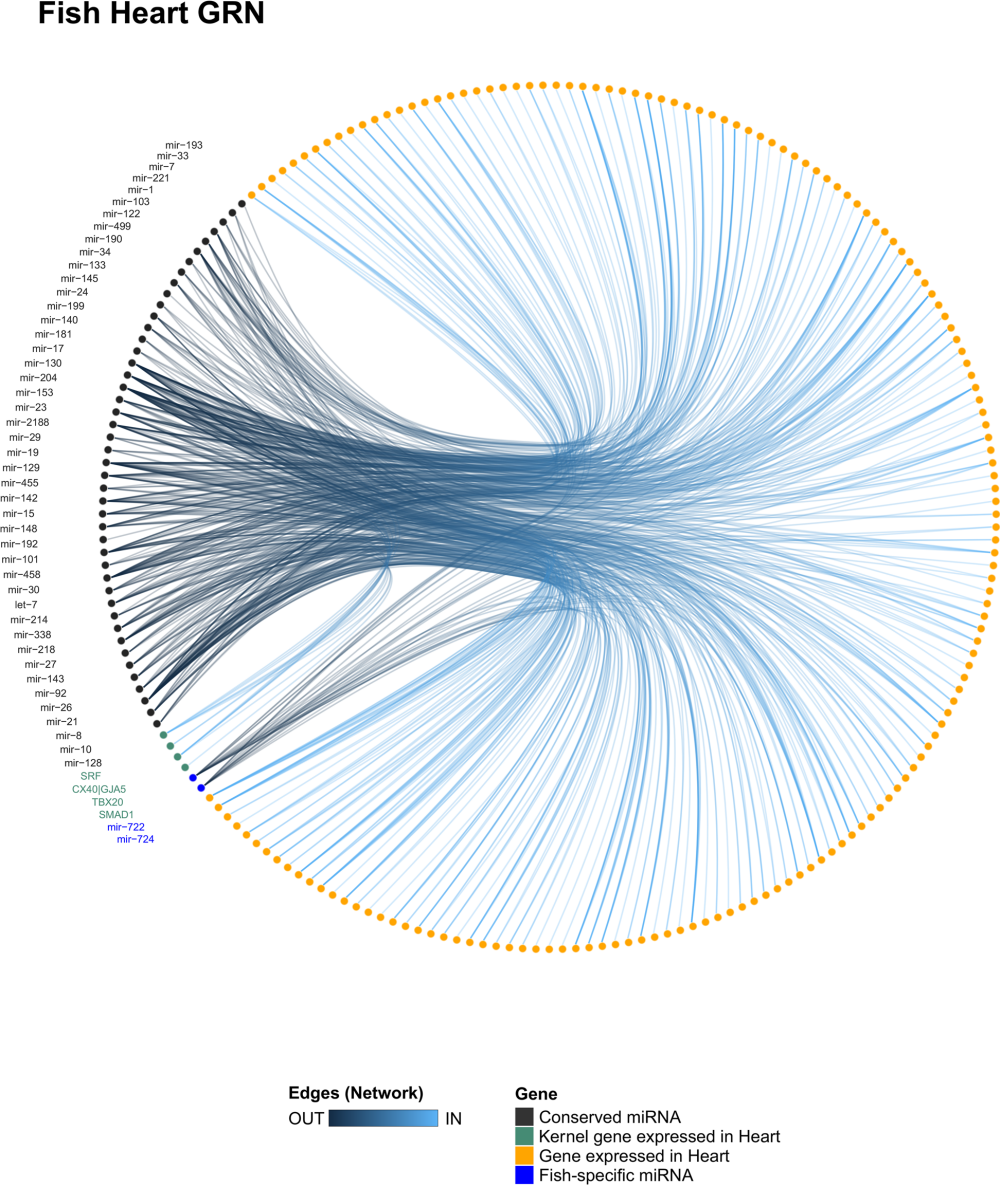
Heart GRN of fishes. The fish heart GRN showing all miR-target interactions detected for the conserved (black) and group-specific miRNAs (blue)

In the fish heart network (Fig. 4), we noticed that the conserved miRNAs miR-8, miR-130, and miR-181 presented a high degree and closeness score (Tables S3 and S4 in Additional file 4). These miRNAs may be acquiring a central role in the network by targeting several genes and helping to fine-tune various biological processes. However, most miRNAs may be acting as peripheral genes in the network, which suggests that they play roles in specific biological processes in the heart of fishes. We detected that miR-26 interacts with SMAD1, which indicates that this miRNA may exert a pivotal role in specific processes, such as cardiomyocyte proliferation, differentiation, and tissue homeostasis in an adult context [34]. Interestingly, we noticed that six conserved miRNAs (i.e., miR-23, -128, -129, -338, -458, and -455) and the fish-specific miR-724 putatively target the gene ENSP00000218867 (SGCG; sarcoglycan gamma), which is a gene related to heart contraction and cardiac muscle development. In this sense, these miRNAs may be acting to control the heart contraction rate observed in fish species, which is lower in fishes than other vertebrate groups [2]. We also detected that miR-8 and miR-722 putatively interact with the gene ENSP00000353408 (MSN; Moesin), which is a gene related to cellular proliferation, suggesting a role for miR-722 and miR-8 in myocyte proliferation. Moreover, we also detected the following validated interactions fish species: miR-145 targeting GATA6 in zebrafish, miR-1 targeting HAND2 in zebrafish, and miR-499 targeting ENSP00000379644 (SOX6; SRY-Box Transcription Factor 6) in zebrafish and Nile tilapia ([35, 36]; Table S2 in Additional file 4), suggesting these miRNAs are important modulators in the heart of fish species.

**Fig. 5:**
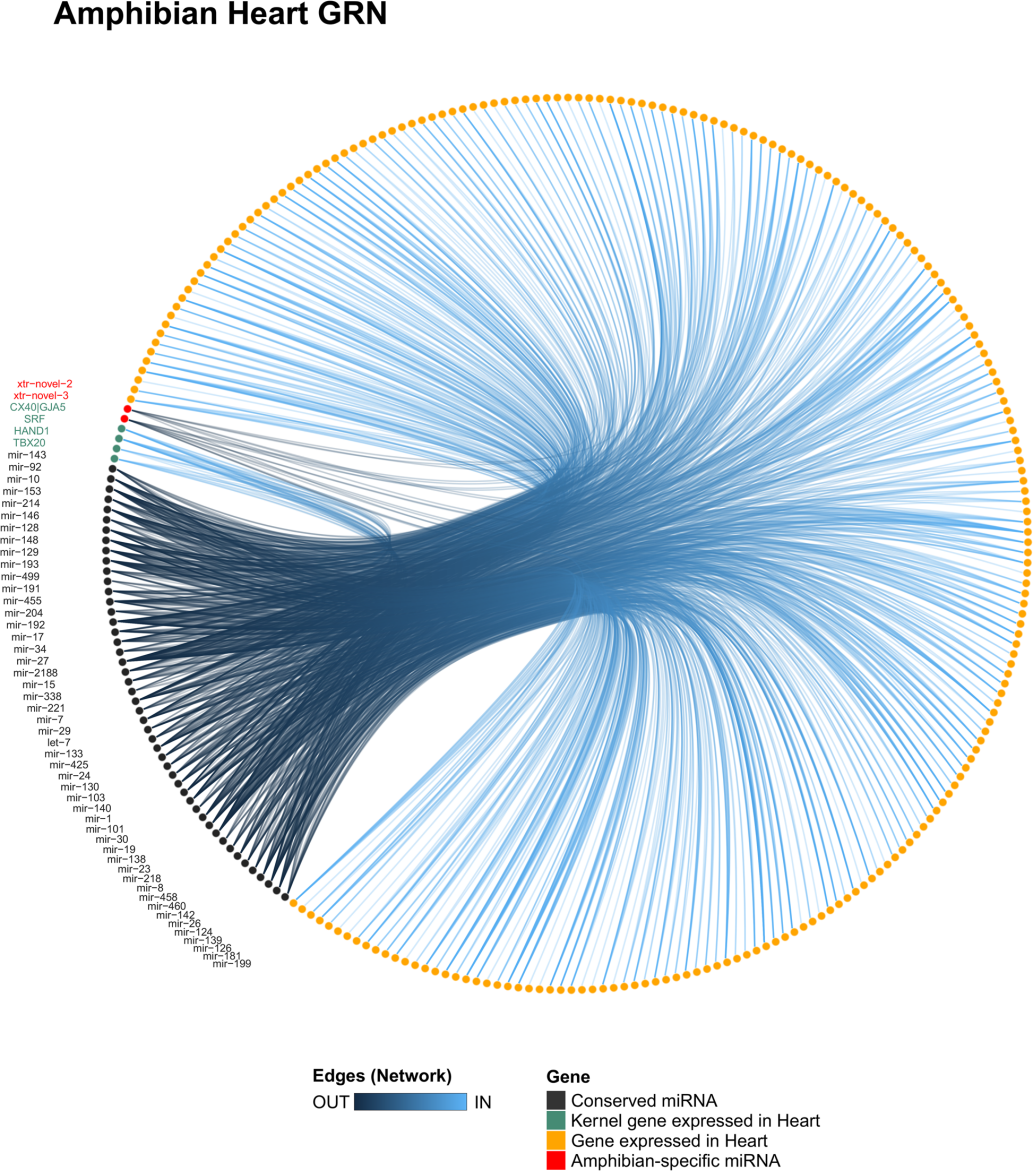
Heart GRN of amphibians. The amphibian heart GRN showing all miR-target interactions detected for the conserved (black) and group-specific miRNAs (red)

In the amphibian network (Fig. 5), we detected that miR-8, miR-19, miR-126, miR-193, and miR-214 presented a high level of degree and closeness score among all miRNAs (Tables S5 and S6 in Additional file 4). Interestingly, these miRNAs were predicted to target the kernel genes of heart GRN, which suggests that their functions may be related to core functions in the heart of amphibians. The miR-129 and miR-221 putatively target HAND1, which indicates that these miRNAs are acting on cardiac cell proliferation [37]. The interactions between those miRNAs and HAND1 were only predicted in amphibians (Table S1 in Additional file 4), indicating that the modulation of expression of HAND1 by miR-129 and miR-221 is occurring specifically in the amphibian heart GRN. The miR-204 was predicted to target TBX20 and ENSP00000353408 (MSN; Moesin), suggesting a role for miR-204 in myocyte proliferation and chamber morphology. Moreover, the miR-338, miR-191, and let-7 were predicted to target CX40, indicating that those miRNAs may be playing roles in the heart contraction rate. Furthermore, we detected pairs of previously validated interactions in the heart such as between miR-1 and HAND2 and miR-128 and ISL1 ([38]; Table S2 in Additional file 4), which shows that both miRNAs may be important regulators in the amphibian heart GRN.

**Fig. 6:**
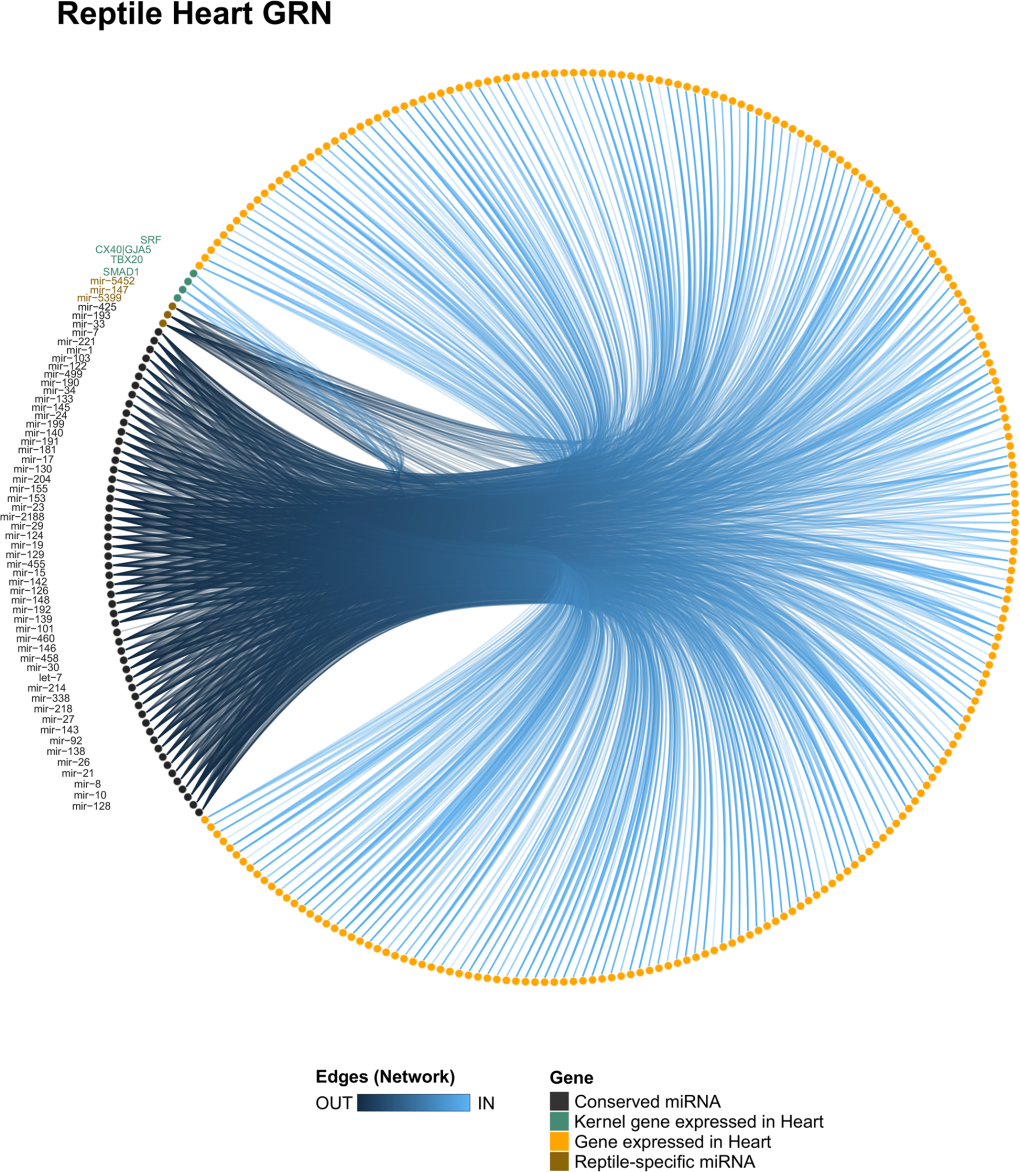
Heart GRN of reptiles. The reptile heart GRN showing all miR-target interactions detected for the conserved (black) and group-specific miRNAs (brown)

In the reptile network (Fig. 6), the conserved miRNAs miR-8, miR-17, miR-101, miR-199, and miR-204 presented a high degree and closeness score, which indicates that these miRNAs may be turning into central genes by interacting with several genes (e.g., kernel and/or peripheral genes; Tables S7 and S8 in Additional file 4). The conserved miR-221 and the reptile-specific miR-5399 were predicted to interact with SMAD1, which suggests that those miRNAs are acting together to modulate cardiomyocyte proliferation and differentiation. The miR-24 and miR-122 putatively target the SRF, which is a gene with a known function in regulating the muscle cell proliferation process [39]. The mir-142 and mir-27 were predicted to target ENSP00000362151 (FOXP4; foxhead box P4), whereas the mir-21 putatively interacts with ENSP00000477817 (PTPDC1; protein tyrosine phosphatase domain containing 1), indicating that both communities may play regulatory roles on general processes of the cardiac cells, such as transcription and dephosphorylation.

**Fig. 7:**
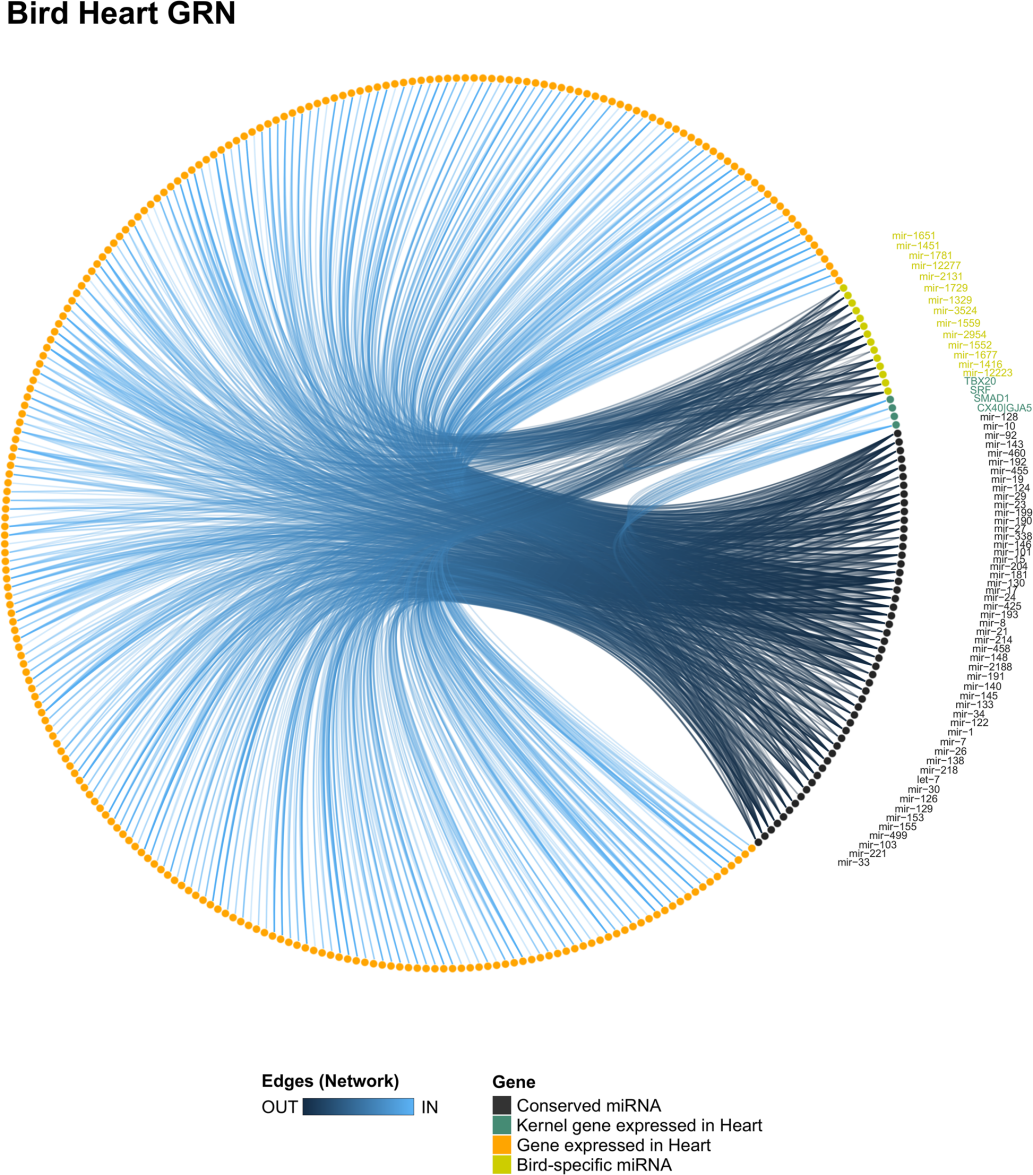
Heart GRN of birds. The bird heart GRN showing all miR-target interactions detected for the conserved (black) and group-specific miRNAs (yellow)

In the bird heart network (Fig. 7), the conserved miRNAs miR-8, miR-15, and the bird-specific miR-1329 presented a higher level of degree and closeness score among all miRNAs (Tables S9 and S10 in Additional file 4), suggesting these miRNAs may be added to the heart network of birds. We noticed that several conserved and bird-specific miRNAs were predicted to target SRF and ENSP00000353408 (MSN; Moesin), which indicates that those miRNAs may be acting together to modulate the cellular proliferation process in the heart of birds. The miR-10 was predicted to interact with ENSP00000218867 (SGCG; sarcoglycan gamma), which is a gene related to heart contraction and cardiac muscle development. Moreover, we identified a validated interaction between miR-1 and HAND2 in chicken (Table S2 in Additional file 4), which indicates that miR-1 may be added in the kernel of bird heart GRN by regulating the cardiomyocyte proliferation process [37].

**Fig. 8:**
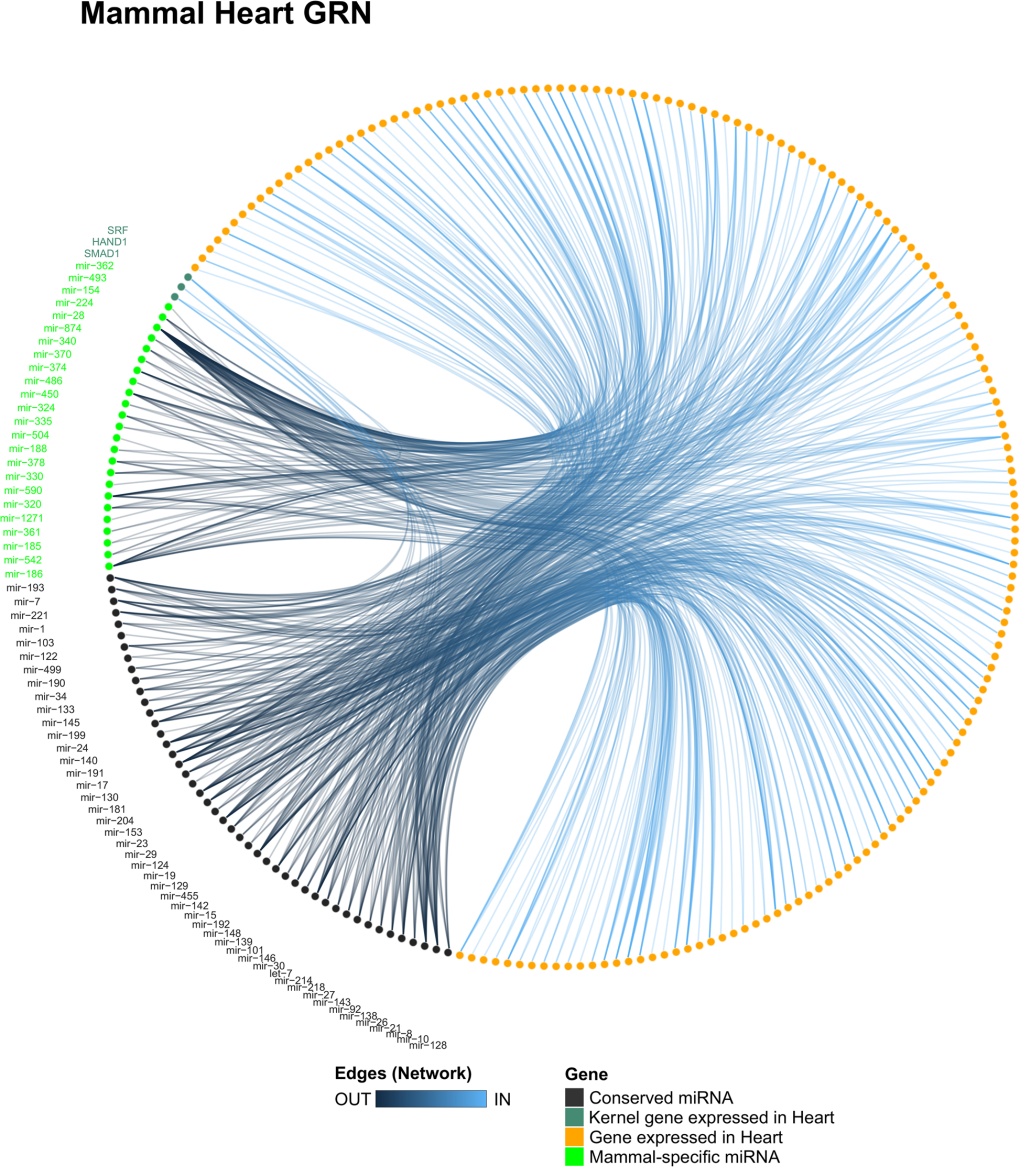
Heart GRN of mammals. The mammal heart GRN showing all miR-target interactions detected for the conserved (black) and group-specific miRNAs (green)

In the mammal heart network (Fig. 8), the conserved miRNAs miR-8, miR-17, and miR-181 presented a high degree and closeness score among all miRNAs (Tables S11 and S12 in Additional file 4), which indicates that these miRNAs may participate in several pathways. Moreover, among the mammal-specific miRNAs, the miR-154 presents a high degree and closeness score, which indicates that this miRNA may be playing a central role in the heart GRN of mammals by targeting several genes, suggesting roles for miR-154 in several pathways of the mammal heart. The conserved miRNAs miR-26 and miR-142 present binding sites in the 3’UTR of SMAD1, suggesting an integrative effort among both miRNAs to possibly modulate the expression of SMAD1 to control the cardiomyocyte proliferation, differentiation, and tissue homeostasis processes. Interestingly, two conserved miRNAs (i.e., miR-133 and miR-192) and few mammal-specific miRNAs (i.e., miR-504, miR-542, miR-590, and miR-1271) were predicted to interact with the gene ENSP00000353408 (MSN; Moesin), which is a gene related to the cellular proliferation process. Moreover, we detected validated interaction between several conserved miRNAs with the kernel genes in mammal species (Table S2 in Additional file 4), whereas the mammal-specific miRNAs miR-675 and miR-483 target SMAD1 and SRF, respectively.

### Comparative analysis of miR-target interactions in the heart GRN of vertebrates

We were able to detect conserved miR-target interactions among heart networks of vertebrate groups in the comparative analysis (Additional file 5). Comparing the fish network with the other groups showed that amphibians, reptiles, birds, and mammals present 72, 145, 105, and 75 conserved interactions, respectively. This reveals that the miR-target interactions in the fish network present lowly similarity to other vertebrate groups, which may be related to the differential morphophysiological traits of its heart. The comparison of amphibians with reptiles, birds, and mammals revealed 232, 156, and 50 conserved interactions, respectively. This suggests that miR-target interactions in the heart of amphibians are more similar to reptiles than to other vertebrate groups, which may reflect a shared morphophysiological trait among these groups. Comparing reptiles with birds and mammals revealed 172 and 338 conserved interactions, respectively. The higher similarity detected in reptiles and birds networks may be related to the fact that both groups constitute a monophyletic clade and share a common evolutionary history. Birds and mammals presented 159 conserved interactions revealing high similarity between groups. These conserved interactions between birds and mammals may be related to the higher heart rate observed in both groups [2].

## Discussion

### Comparative analysis of the heart miRNome of vertebrates

Our large-scale comparative analysis of miRNAs composition and expression breadth in the vertebrate heart revealed a common set of 54 miRNA families with deep conserved expression in groups isolated by millions of years during evolution. We also detected several miRNAs with expression group-specific among vertebrates. Interestingly, a previous comparative analysis of the miRNome of several tissues in agnathans and jawed vertebrates (i.e., hagfish, zebrafish, and mouse) revealed that the heart is more heterogeneous than other organs regarding the miRNA expression profile [21]. Another recent study, comparing miRNAs in several tissues of zebrafish and stickleback, detected a substantial number of evolutionary conserved and several species-specific heart-enriched miRNAs [26], reinforcing the peculiar heterogeneity of miRNAs identified in the heart [21]. In fact, miRNAs are specialized in function by displaying an organ/cell-specific expression patterns [40], which indicates that the heterogeneity of miRNAs may be related to the distinct traits observed in the heart of vertebrates, including variable regenerative capacity [41, 42]. By contrast, similarities in the set of miRNAs may correlate with core heart functions across species, such as blood pumping, electric patterning [2], and other pathways related to the basal metabolism of the cardiac cells.

We noticed that most of the conserved miRNA families expressed in the heart of the 20 species investigated can be traced back to 400-690 Million Years Ago (MYA). Conversely, group-specific families stand for younger miRNA families (Table S2 in Additional file 1). Moreover, the conserved miRNA families, constituted by ancient miRNAs, present an elevated number of putative targets and higher expression levels when compared to group-specific families, constituted by young miRNAs. These observations are consistent with the previous hypothesis that ancient miRNAs show higher expression and regulate a wider range of targets than younger miRNAs [12, 23, 43–46], suggesting that the functional roles of ancient miRNAs are well established in the regulation of heart GRNs. On the other hand, group-specific miRNAs, which are younger, have lower expression levels and low diversity of targets, which indicates that group-specific miRNAs may be modulating specific pathways of the heart GRNs. Nevertheless, during evolution, the group-specific miRNAs can gradually increase their expression levels and acquire new targets, thereby strengthening their relevance for the modulation of regulatory networks. In fact, young miRNAs present lower expression levels and a lower number of targets [47], which may contribute to their slow and gradual integration into GRNs of diverse tissues and its component cells during evolution. Overall, this process could be responsible for the functional enhancement of GRNs by the incorporation of the sharp regulation conferred by miRNAs, including the heart GRNs.

### MicroRNAs and the heart GRN

The heart networks obtained for each group indicate that most of the miRNAs may be acting as peripheral genes in the network, suggesting that miRNAs tend to act in specific biological processes. However, some miRNAs may be considered as central genes and added to the kernel heart GRN of vertebrates. Otherwise, few miRNAs may be added to their group-specific heart GRNs, due to the high number of interactions with genes expressed in the heart, including the kernel genes. In this topic, we discuss the miRNAs that may be playing important functional roles in the heart GRN of vertebrates and should be functionally analyzed in future experiments.

Several of the conserved miRNAs were previously described as cardiac miRNAs, being miR-1, miR-133, miR-499, miR-26, and miR-92 [48, 49]. Other conserved miRNAs were not reported to be cardiac miRNAs but have the potential to be integrated into the heart GRN. On the other hand, little is known about the functional roles of the group-specific miRNAs, but they can distinctively modulate the expression of the core genes of the heart GRN allowing for the establishment of the cardiac biology of vertebrates.

The miR-1 is one of the most expressed miRNAs in the heart of all vertebrate species analyzed, which suggests that miR-1 has key roles in the maintenance and homeostasis processes of the heart of vertebrates. We noticed strong functional evidence for the interactions between miR-1 and GATA4 in mammals, and between HAND2 in zebrafish, *Xenopus*, chicken, and mammals [50]. In this sense, the present data indicate that we can include miR-1 in the heart GRN by targeting HAND2 in a conserved manner for all vertebrate groups and targeting Gata4 in a mammal-specific manner. Furthermore, our prediction analysis revealed a putative interaction between miR-1 and ENSP00000286201 (FZD7; Frizzled Class Receptor 7) specifically in reptiles, birds, and mammals. Interestingly, FZD7 is crucial for normal heart development in frogs and any dysregulation of FZD7 expression may result in heart abnormalities [51]. In this sense, the interactions between miR-1 and FZD7 may help to generate the distinct phenotypes observed in the heart of vertebrates. Moreover, knockout of miR-1 in mice results in postnatal lethality due to abnormalities in the heart [52, 53], which suggests an important functional role for miR-1 during cardiogenesis and homeostasis of heart biology.

For the miR-133, we identified a validated interaction between miR-133 and SRF in mice indicating a role for miR-133 in the heart of mammals and putatively other vertebrates. In fact, a previous report showed that miR-133 regulates SRF and enhances muscle cell proliferation in mammals [39] and another recent report showed that miR-133 promotes cardiac hypertrophy in zebrafish [54]. Another study performed a knockout experiment of the miR-133 locus in mice, which resulted in perinatal lethality due to heart defects [55]. They showed that the heart defects could be associated with an excessive cardiac cell proliferation caused by the absence of miR-133 [55]. Moreover, our prediction analysis revealed that miR-133 may also interact with ENSP00000353408 (MSN; Moesin) in reptiles, birds, and mammals, the MSN is a gene that regulates the cell proliferation process. Moreover, miR-133 participates in heart regeneration in zebrafish [56]. Yin and colleagues showed that an induced elevation of the miR-133 level after injury inhibits the cardiomyocyte proliferation [56], whereas the absence of miR-133 enhances the proliferation process of cardiomyocytes. All these data indicate that miR-133 is an important modulator of cardiomyocyte proliferation and may integrate the kernel heart GRN of vertebrates.

The miR-499 is known to participate in slow-twitch muscle fiber specification from fish to mammals [35, 36, 57], and to be involved in cardiac cell differentiation and homeostasis [58, 59]. Our results revealed that miR-499 may be targeting ENSP00000379644 (SOX6) in 10 species belonging to all groups of vertebrates. In fact, the interaction between miR-499 and SOX6 was validated in zebrafish, Nile tilapia, and mouse [35, 36, 57, 59]. In this sense, miR-499 may be playing roles in fiber type specification of cardiac tissue in all vertebrates.

We detected that members of the let-7 family are highly expressed in the heart and let-7 can present a potential function in the heart GRN of mammals, once we detected a validated interaction with BMP4 in humans and predicted a conserved interaction with HAND1 in all mammal species. Interestingly, a study analyzing miRNAs expressed during mouse heart development detected that let-7 targets HAND1 and may play key roles in the heart development network of mammals [60]. Moreover, functional and bioinformatics experiments demonstrated that aberrant expression of let-7 members is related to cardiac diseases and defects in heart development (reviewed by [61]), and that let-7 is required for cardiomyocyte maturation from stem cells by acting on pathways related to metabolism, cell size, and force contractility [62]. In this sense, let-7 may be included in the heart GRN of mammals due to their interactions with the kernel genes.

For the members of the cluster mir-17/92, which comprises four families (i.e., miR-17, miR-18, miR-19 and miR-92; [63]), our target prediction pipeline detected that miR-17 and miR-19 targets the gene ENSP00000351905 (TGFBR2; Transforming Growth Factor Beta Receptor 2) in a conserved manner in all vertebrates, whereas the miR-92 targets HAND1 in a mammal-specific manner. In fact, knockout experiments in mice revealed that the miR-17/18/19a/20a/19b-1/92a-1 locus deletion resulted in perinatal lethality due to heart defects [64, 65]. In this sense, miR-92 may be included the heart GRN of mammals associated with cardiac cells proliferation and heart morphogenesis by targeting HAND1 [37, 66], whereas miR-16 and miR-17 may be added as modulators of heart looping and heart development in all vertebrates by targeting TGFBR2.

The miR-8 presented a putative functionality as a central gene in the heart network of vertebrates (i.e., high degree and closeness score in all groups). In fact, any dysregulation in miR-8 expression may be related to cardiovascular diseases (reviewed by [67]). The miR-8 presents a validated interaction with the gene ENSP00000354487 (ZEB1; Zinc Finger E-Box Binding Homeobox 1) in mouse and human [68], which is a gene expressed in several organs, including the heart, and enhances cell differentiation. Moreover, we predicted a conserved interaction between miR-8 and ZEB1 in 9 species of vertebrates. Another pathway that miR-8 may be acting on the heart GRN of vertebrates relies on the conserved interaction detected between miR-8 and the kernel gene SRF, which is a gene that enhances cardiomyocyte differentiation, and other genes that are related to cell proliferation and general processes of the cell (e.g., ENSP00000353408 - MSN, ENSP00000011619 - RANBP9, and ENSP00000366897 - KLF12). In this sense, miR-8 may be an important modulator of the myocyte proliferation and differentiation in the heart GRN of vertebrates.

The miR-26 is a conserved miRNA that may be added to the kernel due to the validated interaction with SMAD1 detected in humans and the conserved interaction predicted in 10 species from the 14 analyzed in the present study. In fact, miR-26 may play pivotal roles in the heart (reviewed by [69]). In this sense, miR-26 should be the focus of further experiments to elucidate its functional roles in the heart GRN of vertebrates.

The conserved miRNA miR-130 may be acting as a peripheral gene and can be associated with heart morphology by interacting with the gene ENSP00000351905 (TGFBR2) in a conserved manner. In fact, overexpression of miR-130 causes ventricular wall hypoplasia and ventricular septal defect [70], which suggests that this miRNA is acting on the specific process controlling the myocyte size and cardiac septation.

Looking at the group-specific miRNAs in the fish heart GRN, we noticed that the miR-724 may be regulating the gene ENSP00000218867 (SGCG), which suggests roles on the heart contraction rate, whereas the miR-722 is putatively targeting the gene ENSP00000353408 (MSN), which indicates roles in the myocyte proliferation process.

The reptile-specific miR-5399 was predicted to target SMAD1, which indicates a role, specific to reptiles, for this miRNA in the processes of cardiomyocyte proliferation, differentiation, and tissue homeostasis in an adult context [34].

Alongside the bird-specific miRNAs, we were able to detect that the miR-1552 and miR-12223 may be acting together on the gene ENSP00000353408 (MSN) and that miR-1552 is possibly targeting SRF, which indicates that both miRNAs may be acting on controlling the cellular proliferation process in the cardiac cells of birds.

The mammal-specific miR-154 was predicted to target several genes expressed in the heart of mammals, including a few kernel genes. Such interactions indicate that miR-154 may be acting on controlling the myocyte proliferation processes and is playing central roles in the mammal heart GRN. In fact, several studies showed that miR-154 controls the myocyte proliferation, fibrosis, and cardiac remodeling processes in the heart of mice [71–73]. In this sense, the miR-154 is an important modulator of cardiac homeostasis in mammal heart GRN.

Overall, we were able to show that several miRNAs may be included in the heart GRN of vertebrates and noticed that most of the interactions detected are supported by previous experimental data. Moreover, we discussed the putative importance of the conserved and group-specific miRNAs in cardiogenesis and their feasible function in heart morphophysiology maintenance and evolution. However, further rigorous quantitative analysis and functional experiments are necessary to validate the several miRNA-target interactions detected by our pipeline.

One limitation of the pipeline applied in the present study is related to the fact that we only used interactions available from Human data in the STRING database due to the higher number of interactions available for this species when compared to other species. We did not test for bias related to other gene interactions taking place specifically in other vertebrate species. In this sense, our pipeline may be unavailable to detect interactions between proteins specifically in each group. However, we believe that this issue may not heavily disrupt the results obtained and discussed in the present study once we focused our analysis on miRNA-target interactions.

### MicroRNAs in the evolution of heart GRN

Although the kernel of heart GRN is well established [74], the evolutionary aspects that drive the morphophysiological differences and similarities among vertebrates remain unclear. Remarkably, changes in gene expression regulation are considered the core of phenotypic differences among species [9, 75]. GRNs evolve at both cis and trans levels [76], and the identification of cis-regulatory modules (CRMs) is essential to understand GRN evolution. Considering that transcription factors act at the trans level of a GRN and are the core components of a GRN (i.e., the kernel), almost no changes in their interactions are observed during evolution. Thus, the CRMs are the main components driving the final phenotypes that resulted from a GRN. In this sense, it is possible that miRNAs act as CRMs to shape the evolution of GRNs governing the morphophysiological differences observed in the heart of vertebrates [7].

As discussed, the co-opting model, which proposes that the appearance of novelties during evolution is based on the co-opting of new components in pre-existing GRNs [77–79]. For example, a comparative analysis based on genomic data suggested that muscle, immune and neuron cells evolved by the co-opting of a pre-existing genetic regulatory system common to these cell types [78]. Another recent report suggested that GRNs produce a similar phenotype in distinct contexts by retaining a set of core components but with differences in the CRM components and their connections [80]. These differences in CRMs reflect the changes that accumulate in GRNs during evolution through the co-opting process. In this sense, our data suggest that miRNAs can be integrated into the heart GRN by the co-opting model. Then after the inclusion of miRNAs in the heart GRN, the expression level of miRNAs and its targets could shape the appearance and maintenance of novel phenotypic traits in the heart of vertebrates along evolution.

Another key point on the evolution of heart GRN through the inclusion of miRNAs can be related to the fact that any disturbance of miRNA expression should lead to heart defects and abnormalities [16, 17], which can be associated with the specific action of miRNAs on their targets. It is known that a single gene interaction can impact the phenotype without critical pleiotropic effects, which is a parsimonious mechanism for the GRN evolution [76]. In fact, a single miRNA-target interaction can be responsible for a specific phenotypic abnormality, but all the miRNA-target interactions are disrupted at any level [29]. Thus, it indicates that the entire miRNA network is crucial to normal development, homeostasis, adaptation, and regeneration and the sum of all the various interactions may be directly related to the evolutionary process regarding the shape of heart tissue.

In summary, we hypothesize that the core genes of the heart GRN are important modulators of the heart development, acting as “drivers”, whereas miRNAs are the fine-tuning modulators, acting as “passengers” of the heart GRN, which can confer robustness to the network output and lead to the unique biological traits of cardiac cells in each species.

## Conclusions

Here, we present a comprehensive annotation and comparative analysis of miRNAs expressed in the heart of 20 species representative of all major vertebrate groups. We identified 54 miRNAs with a conserved expression profile and dozens of miRNAs with a group-specific expression profile. The set of conserved and group-specific miRNAs may play roles regulating heart GRN and architecture providing new insights into the evolutionary process underlying the heart phenotypic diversity in vertebrates.

We showed that conserved miRNAs may act distinctively in the heart GRNs of vertebrates, whereas some of the group-specific miRNAs may be playing singular roles in the heart of groups they are being expressed. Our data indicate that some miRNAs may be playing central roles in the heart GRN, by acting in several processes, whereas most of the miRNAs are participating as peripheral genes, by acting in specific pathways.

Future studies can be designed to analyze specific populations of cells beyond heart tissues to expose the biological bases underlying tissue cell diversity and identity. Furthermore, evolutionary time course gene expression studies during development will likely follow a similar workflow to fully disentangle the developmental programs that govern the vertebrate organismal diversity. These developmental studies will be helpful to improve knowledge about heart GRN evolution and will lead to insight into the addition of miRNAs in the heart evolution process of vertebrates.

We have developed a computational workflow that can identify and refine GRNs by unveiling miRNAs with the potential to govern specific biological processes. Moreover, our workflow can help to improve knowledge about heart-disease pathways enabling comparison of datasets from affected and non-affected cells. This can contribute to deciphering the miRNA-target interactions and help to identify new candidates for miRNA-based therapeutics against cardiovascular diseases, a leading cause of mortality worldwide.

## Methods

### Sampling and RNA extraction

Heart samples of *Oreochromis niloticus* (Nile tilapia fish), *Xenopus laevis* (African clawed frog) and *Tropidurus torquatus* (lizard), were obtained from Royal Fish (Jundiaí, SP, Brazil), Lee lab (Vanderbilt University, Nashville, USA) and EvoDevo Lab (USP, Ribeirão Preto, SP, Brazil), respectively. The animals were sacrificed by an overdose of MS-222 anesthetic (50 mg/L tricaine-methanensulfonate; Sigma-Aldrich) for sampling. The tissues were freshly removed from animals, washed in a salt solution (0.9% of NaCl) to clean out the blood, frozen directly in liquid nitrogen, to avoid RNA degradation, and stocked at -80^∘^C until use. The RNA extraction was performed by using the TRI Reagent *Ⓡ* (Sigma-Aldrich) following the manufacturer’s instruction. To avoid genomic DNA contamination, all samples were treated with DNase I (Thermo Fisher Scientific) by using the standard protocol. The quantification and contamination level was measured by absorbance at NanoDrop1000 (Thermo-Scientific). RNA integrity was assessed by using 2100 Bioanalyzer (Agilent) equipment, and only samples with RIN higher than 7 were used to perform the small RNA sequencing. The animals were handled under the approval of the local ethics committee (CEUA - Comissão de Ética no Uso de Animais, protocols numbers 352/11 and 774/15).

### Small RNA library, sequencing and datasets

The high-quality RNA was used for library construction from each sample at LC Sciences company (Houston, USA) by using the Illumina Truseq Small RNA Preparation kit (Illumina) according to the manufacturer’s guide. The libraries were sequenced each in one lane of Illumina GAIIx platform. The raw reads were used in the subsequent data analysis. Additionally, we retrieved small RNA sequencing data of heart samples from other 17 species of vertebrates available at SRA and ENA databases (see Table S1 in Additional file 6 for details of datasets). Thus, the final working heart miRNA-seq dataset comprised 20 vertebrate species. For those datasets with two or more biological replicates, we selected the replicate with the higher quality.

### Processing, mapping and miRNA identification

We applied a common pipeline workflow to all datasets analyzed in the present study, which is described below and summarized in the Additional file 7.

We checked the quality by using FastQC and applied a quality filtering to remove low quality reads by using fastq_quality_filter script from FASTX_toolkit with a second round of quality check when needed. Then, we excluded reads smaller than 18 nt, reads that presented “N” base in its sequence, and reads with no traces of adapter sequence. Then we trimmed the adapter sequences by using fastx_clipper for the single-end sequenced datasets. We matched the first 8 bases of the adapter sequence specific for each library as indicated in Table S2 in Additional file 6. Due to lack of information regarding the adapter sequences at the articles and/or project pages at databases (SRA and ENA), we identified the adapter sequences by aligning the first two hundred reads and checking if the putative adapter sequences were part of Illumina’s preparation kit. Only Juanchich et al. [81] have provided the adapters sequences used. We have not performed the quality check and clip adapter for the zebra finch dataset, because we developed a Python script to acquire the miRNAs sequences and un-normalized reads values for each miRNA previously detected by [82] using the available data at the supplementary files of the original article.

We used miRDeep2 tool for mapping of reads and for miRNA identification and annotation [83] by following the steps described below. The reads were mapped by using the mapper.pl module of miRDeep2 that uses Bowtie tool for mapping. We mapped reads of each species dataset against its own genome, except for *Tropidurus torquatus*, that were mapped against the genome of the closely related species, *Anolis carolinensis*, due to unavailability of *T. torquatus* genome sequence (see Table S3 in Additional file 6 for more details).

The known and novel miRNAs were identified by using the miRDeep2.pl module. For known miRNA identification, we combined the miRNA data available and curated at miRBase (www.mirbase.org/; release 21; [84]) and MirGeneDB (http://mirgenedb.org/; version 2.0; [85, 86]), but using the curated miRNAs annotated for metazoan only. For notation, we considered the miRNAs available for its own species. In the case where the species had no previous miRNA annotation available, we referred the annotation to the closely related species. In addition, miRDeep2 predicted putative novel miRNAs based on hairpin-like secondary structure of the precursor sequence and site cleavage presence for Drosha and Dicer enzymes.

When reads presented a multi-mapping characteristic, we identified the supposed precursor loci of transcription origin by applying the Unique Weighting method within the ShortStack tool [87]. This strategy replaces the random placement of reads generated within Bowtie mapping and considered in miRDeep2 results. The Unique Weighting method avoids the bias of strand selection that resulted from the random placement of multi-mapping reads, which can distort results and the downstream analyses. We excluded those loci detected by miRDeep2 pipeline that did not present any unique reads in the ShortStack analysis. Then, we used the ShortStack mapped read values as library size for the subsequent analysis. We normalized the data by applying the TMM method (trimmed mean of M values; [88]) available at edgeR package from Bioconductor (https://bioconductor.org/) that uses R statistical programming (http://www.R-project.org), through considerations of [89] about optimization of miRNA-seq normalization methods.

### Characterization of heart miRNA families

Considering that miRNAs sharing an identical seed sequence are grouped as a family, as they are usually predicted to act redundantly on a set of common targets. In fact, phenotypic analysis in several organisms supports this notion by showing that knockout of all members of a family exhibits a more severe phenotype than deletion of a single miRNA [64, 90, 91]. In this sense, we developed an in-house Python script to classify miRNA families based on the MirGeneDB annotation [85, 86], which considers that all members of a family had a similar evolutionary history by sharing a common ancestor precursor sequence. Thus, our workflow used the precursor sequence of the miRNAs identified to BLAST search against MirGeneDB sequences and considered only hits with at least 80% of coverage. If the precursor has no hit against MirGeneDB, we also perform a similar BLAST search against miRBase sequences and manually check the BLAST results to perform the miRNA family annotation. If there are no hits against miRBase, we assigned no family and considered the miRNA as a putative novel miRNA and classified it as species-specific.

After family identification, we developed two in-house Python scripts: (1) one to characterize the miRNA family abundance and expression level in each species; and (2) another to detect the age of heart miRNA families expressed in vertebrates based on the estimated evolutionary age of gain and loss of animal miRNA families following the data reported by [86].

### Comparative analysis

We performed a large-scale comparative analysis of vertebrate heart miRNAs landscape by comparing qualitative data. In this sense, we grouped miRNAs based on the similarity of mature sequences, which means that miRNAs with identical mature sequences were grouped as a single miRNA (i.e., miRNAs named as locus “-1”, “-2” or “-3”, were grouped together). Any differences at the 3’ end of mature sequences assigned them to a distinct miRNA group (i.e., miRNAs named as “a”, “b”, and “c”, were grouped as different miRNAs). This analysis was conducted by using an in-house Python script. Furthermore, we checked for evolutionary conserved or group-specific expression profiles of miRNA families using as criteria the manifestation of the expression of any member of the family. We considered the miRNA family as conserved, if at least one member of the miRNA family is expressed in all groups of species analyzed. On the other hand, we considered the miRNA family as group-specific, if the miRNA family has expression exclusively on that group, independently if the miRNA family is present or absent in other vertebrate genomes. Then, we applied Fisher’s exact test to check the statistical significance of intersections among datasets by using the SuperExactTest package in R environment [92].

### Target prediction

Our target prediction step was performed using the combination of results generated by TargetScan (http://www.targetscan.org/vert_71/) and miRanda (http://www.microrna.org/) to improve the performance of the prediction analysis [93]. For TargetScan, we used its stand-alone scripts to perform the prediction of target sites followed by a step to calculate the context-score for each target site, we used both scripts within default parameters. For miRanda, we set the parameter “-strict” to avoid the detection of target sites containing gaps or non-canonical base pairing in the seed region. We retrieved the 3’UTR sequences available for each species at BioMart from Ensembl. Moreover, we complemented the 3’UTR annotation for genes with uncharted 3’UTR by using the polyadenylation consensus sequence AAUAAA to predict the 3’UTR of the gene. The sequence AAUAAA is highly conserved and found in almost 90% of all known sequenced polyadenylation signals [94]. We matched the closest AAUAAA sequence to the stop codon to avoid false-positive rates. Moreover, if the predicted 3’UTR was longer than 2,500 nts, we considered the second consensus sequence AUUAAA to predict the 3’UTR [94].

After the target prediction step, we filtered the list of putative targets by keeping only genes expressed in the heart organ of each species by using heart transcriptome data available at the SRA and ENA database (see Table S4 in Additional file 6 for more details). The transcriptome analysis of heart datasets was performed using the Kallisto algorithm [95] and only genes with TPM higher or equal to 1 were considered expressed in the heart and kept as putative targets. The combinatorial and filtering steps of target prediction were performed by using an in-house Python script, which generates the results for the members of the conserved and group-specific families of miRNAs for each species. Unfortunately, due to the unavailability of heart transcriptome for a few species (i.e., electric eel, salmon, rainbow trout, *X. laevis*, zebra finch, and rabbit), we only used 14 vertebrate species in the miRNA target prediction analysis. However, our analysis kept at least one representative species for each vertebrate major group.

### Heart GRN analysis

After the target filtering step, we detected the human orthologous genes from each species using the BioMart tool from Ensembl and designed the networks based on the interactions available in the STRING database (v11.0; [96]). To ensure that only validated interactions in the STRING database were considered in the subsequent analysis, we only kept interactions with experimental scores higher than 0 and combined scores higher than the experimental score (i.e., only interactions with experimental evidence and at least one more evidence were kept for downstream analysis). Moreover, we only kept genes with conserved expression among samples and that presented at least one interaction with one or more genes of the kernel of heart development [7]. In the groups containing more than one species in the GRN analysis, we applied a conservation filter to keep only conserved interactions. For instance, we kept interactions present in at least two of the three fish species and kept interactions present in at least four of the eight mammal species.

We further performed a centrality analysis by measuring the node degree and closeness centrality for the GRN generated to each group. The centrality measures are calculated based on the interactions of nodes in the network and indicate when a node is central or peripheral. The detection of central nodes unveil genes that are modulators of several biological processes in the network, whereas the detection of peripheral nodes reveals genes that are acting on specific biological processes in the network. We also performed community detection by using the Walktrap algorithm [97]. A community is defined as a subset of nodes where its connections are denser than the connections with the other nodes of the network. The community detection helps to unveil relationships between nodes that may be acting in similar biological processes in a network. Then, we manually check the differences and similarities in the interactions in the GRN of vertebrate groups. Moreover, to complement the network analysis and confirm the interactions observed, we checked for strong validated interactions among miRNAs and their putative targets in the miRTarBase (release 7.0; [98]) and scientific literature. The GRNs were constructed using in-house Python scripts whereas the charts and analysis of the network, centrality analysis, and community detection were designed using the igraph and ggraph packages in R environment [99, 100].

We also compared the networks by calculating the Pairwise Jaccard similarities of miR-target interactions [101] and performing a pairwise network alignment to check for conserved miR-target interactions among vertebrate groups.

## Supplementary Information

**Additional file 1** Supplementary data in xls format.

**Additional file 2** Supplementary data in xls format.

**Additional file 3** Supplementary data in pdf format.

**Additional file 4** Supplementary data in xls format.

**Additional file 5** Supplementary data in xls format.

**Additional file 6** Supplementary data in xls format.

**Additional file 7** Supplementary data in pdf format.

## Data Availability

The heart miRNA-seq data of Nile tilapia, African clawed frog and lizard are available in the NCBI BioProject database under the accession number PRJNA560566. The heart miRNA-seq data from all other vertebrate species are available in the NCBI SRA database (accession numbers SRR1554476, SRR866605, SRR2473346, SRR1231994, SRR553599, SRR553594, SRR553589, SRR6662685, SRR4048260, SRR553584, SRR553579, and SRR553574) and EMBL-EBI ENA database (accession numbers SRR1736653, SRR1047498, SRR2062562, and SRR3587077). The heart RNA-seq data from vertebrate species analyzed are available in the NCBI SRA database (accession numbers SRR2013387, SRR391681, SRR2054794, SRR1524252, SRR579563, SRR2515151, SRR579558, SRR306714, SRR306730, SRR306749, SRR2226636, SRR388744, SRR087419, SRR306768, SRR306782, SRR306849).

## Abbreviations

miRNA: microRNA
3’UTR: Three prime untranslated region
GRN: Genetic regulatory network
MYA: Million years ago
BMP4: Bone morphogenetic protein 4
TBX5: T-box transcription factor 5
TBX20: T-box transcription factor 20
HAND1: Heart and neural crest derivatives expressed 1
HAND2: Heart and neural crest derivatives expressed 2
GATA6: GATA binding protein 6
GATA4: GATA binding protein 4
SMAD1: SMAD family member 1
GJA5/CX40: Gap junction protein alpha 5
SRF: Serum response factor
ISL1: ISL LIM homeobox 1
SGCG: Sarcoglycan gamma
MSN: Moesin
SOX6: SRY-box transcription factor 6
FOXP4: Foxhead box P4
PTPDC1: Protein tyrosine phosphatase domaincontaining 1
FZD7: Frizzled class receptor 7
TGFBR2: Transforming growth factor beta receptor 2
ZEB1: Zinc finger e-box binding homeobox 1
RANBP9: RAN binding protein 9
KLF12: Krueppel-like factor 12
