# Supplementary figures and images for: A comparative analysis of heart microRNAs in vertebrates brings novel insights into the evolution of genetic regulatory networks
Source: BMC Genomics. 2021 Mar 4;22:153. doi: 10.1186/s12864-021-07441-4 (PMC7931589; doi:10.1186/s12864-021-07441-4)

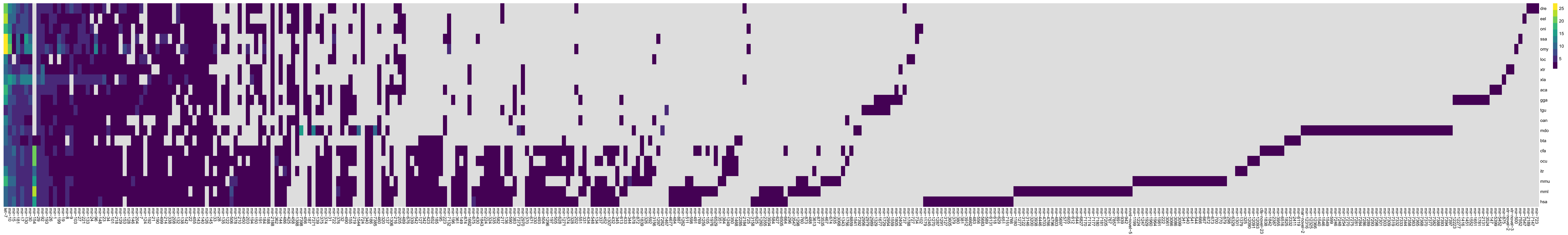

Supplement: Supplementary file 3 — Additional file 3 Supplementary data in pdf format. [file 12864_2021_7441_MOESM3_ESM.pdf]
