# Supplementary material for: A comparative analysis of heart microRNAs in vertebrates brings novel insights into the evolution of genetic regulatory networks
Source: BMC Genomics. 2021 Mar 4;22:153. doi: 10.1186/s12864-021-07441-4 (PMC7931589; doi:10.1186/s12864-021-07441-4)
Supplement: Supplementary file 5 — Additional file 5 Supplementary data in xls format. [file 12864_2021_7441_MOESM5_ESM.pdf]

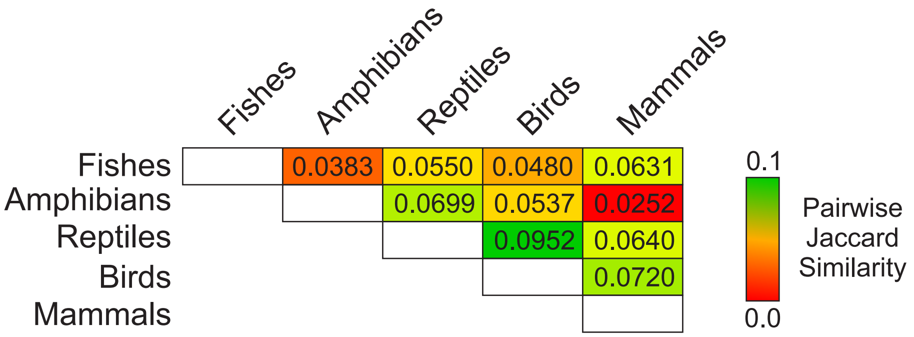

Supplementary figure S1. Pairwise Jaccard Similarity of miR-target interactions detected in each group of vertebrates.

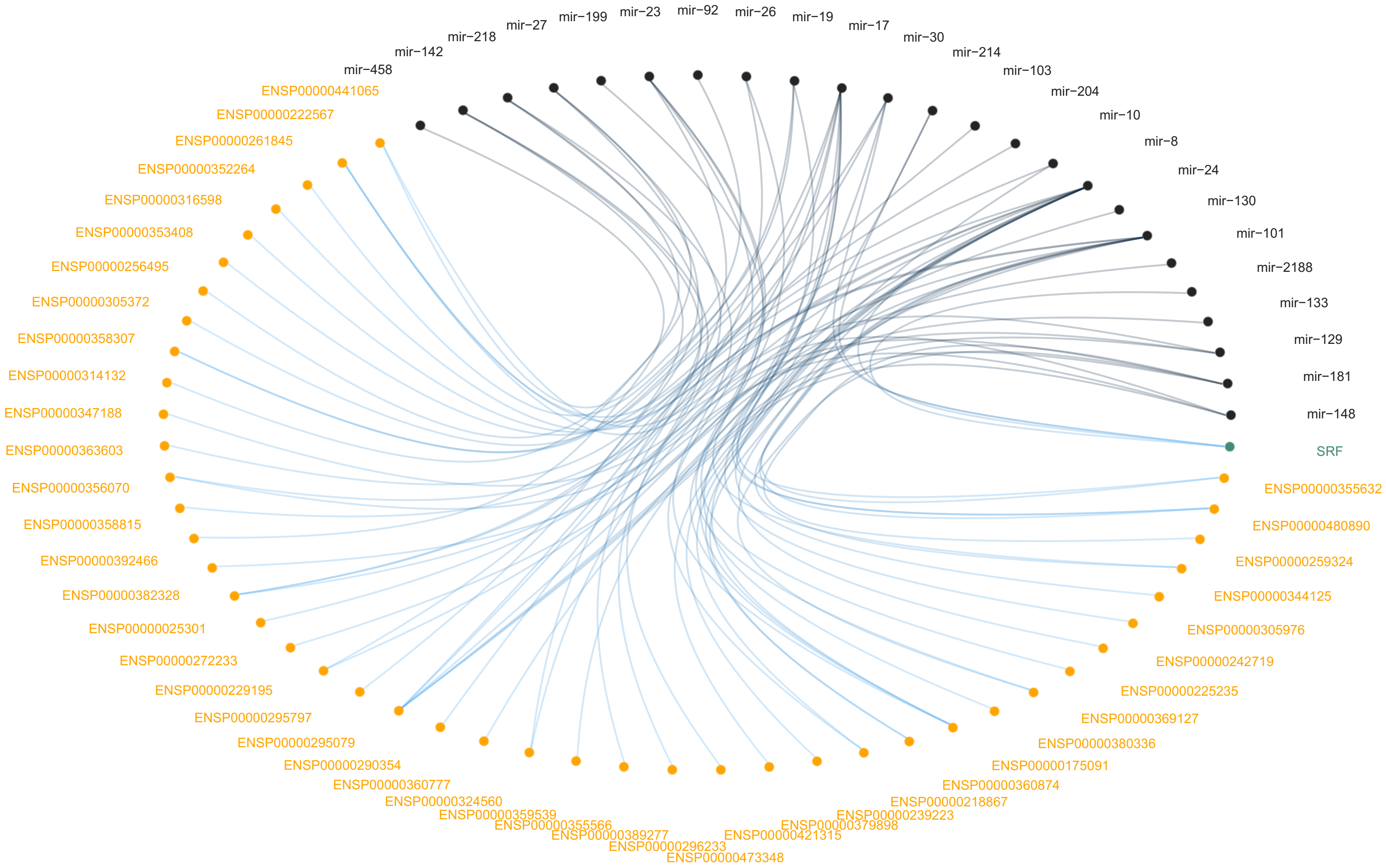

Fishes X Amphibians

Number of conserved interactions: 72

Supplementary figure S2. Pairwise alignment of miR-target interactions detected in heart of fishes and amphibians.

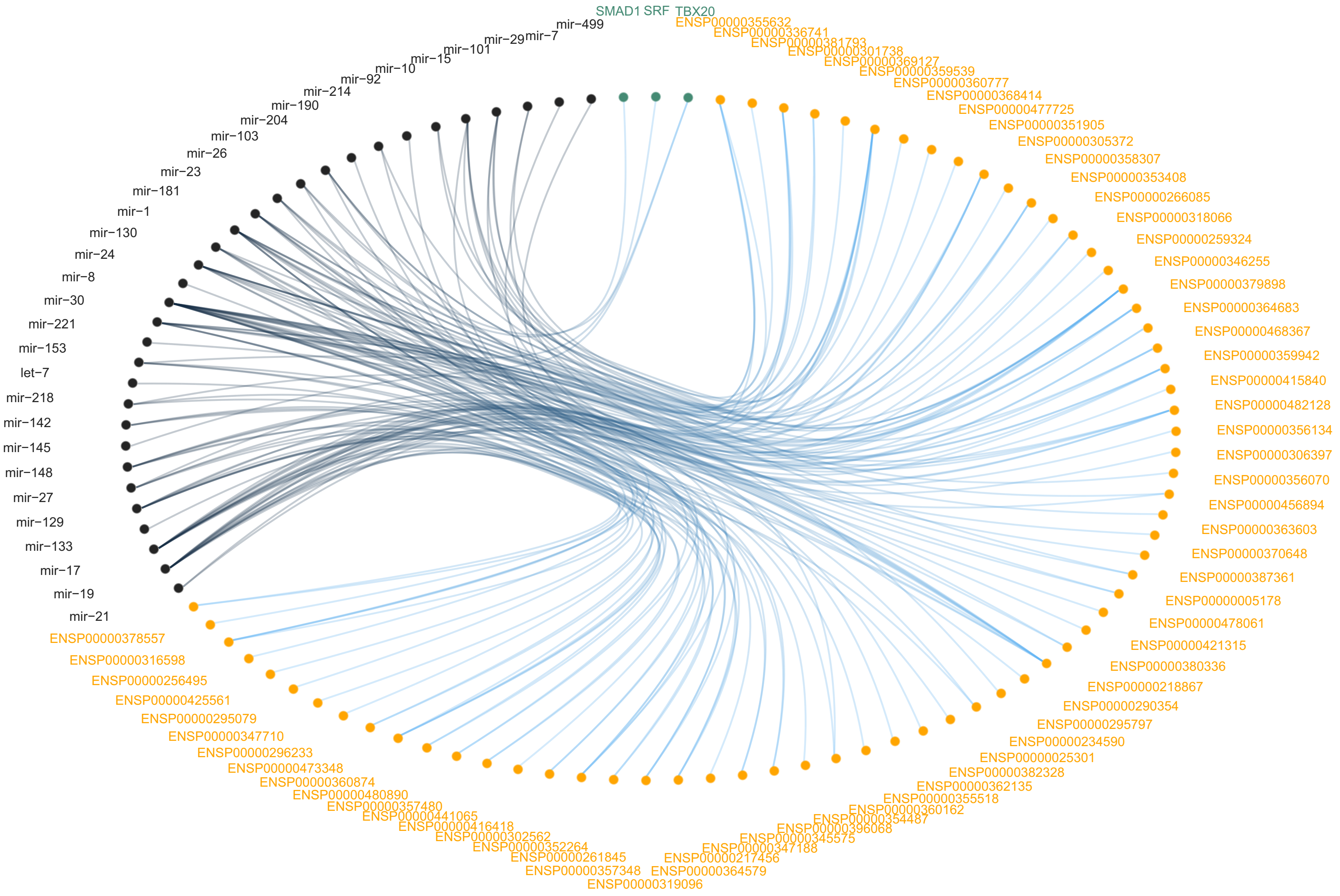

Fishes X Reptiles

Number of conserved interactions: 145

Supplementary figure S3. Pairwise alignment of miR-target interactions detected in heart of fishes and reptiles.

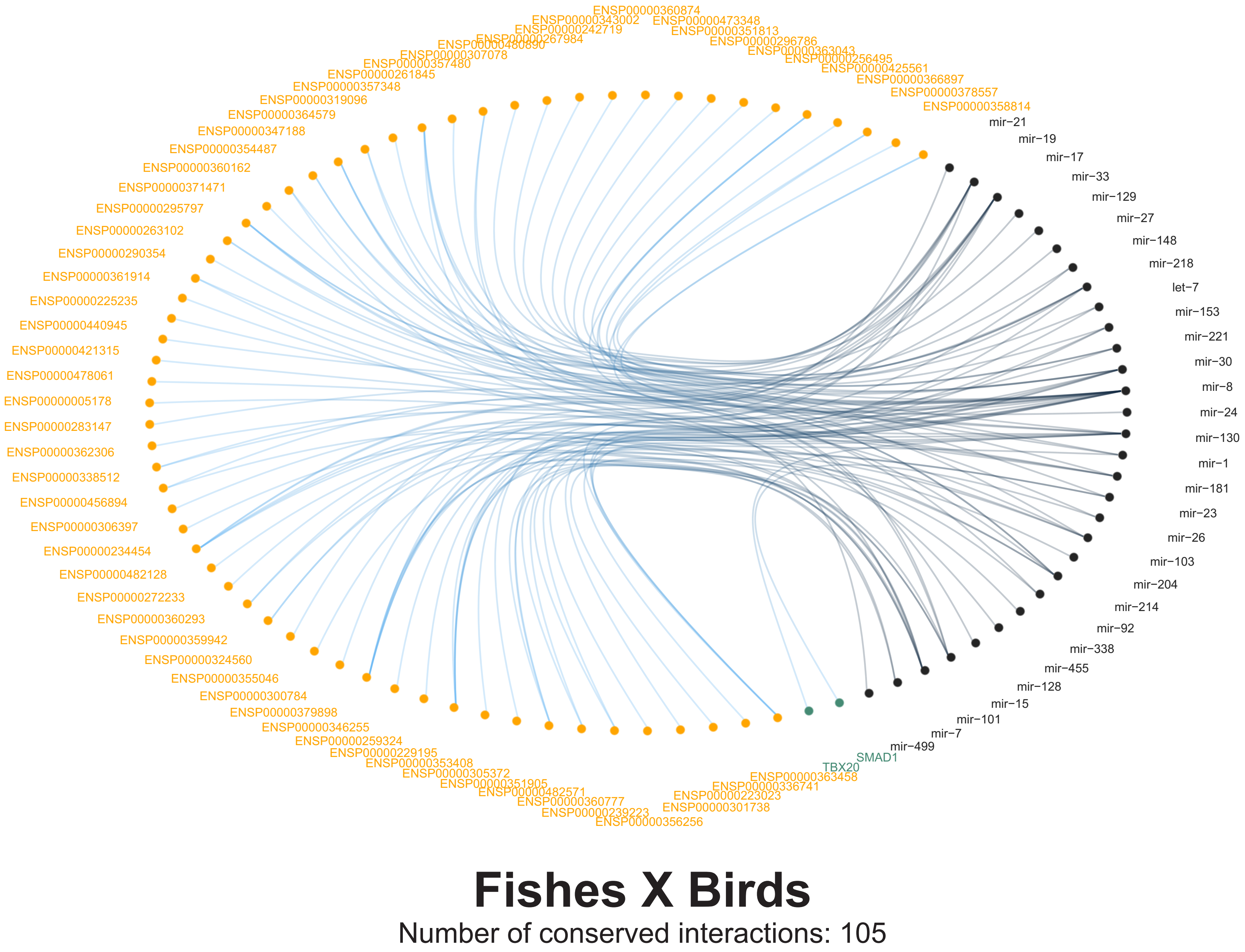

Supplementary figure S4. Pairwise alignment of miR-target interactions detected in heart of fishes and birds.

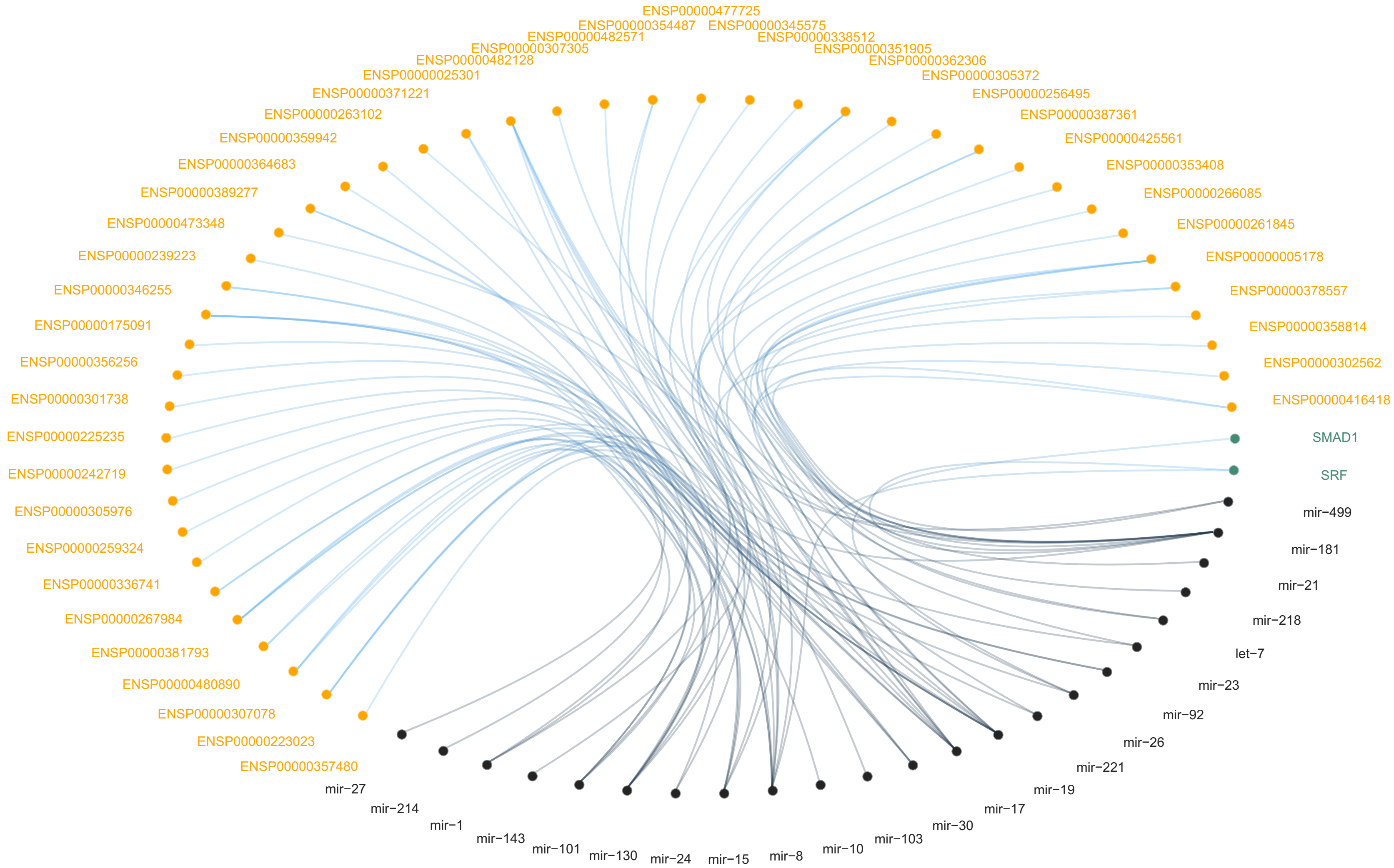

Fishes X Mammals

Number of conserved interactions: 75

Supplementary figure S5. Pairwise alignment of miR-target interactions detected in heart of fishes and mammals.

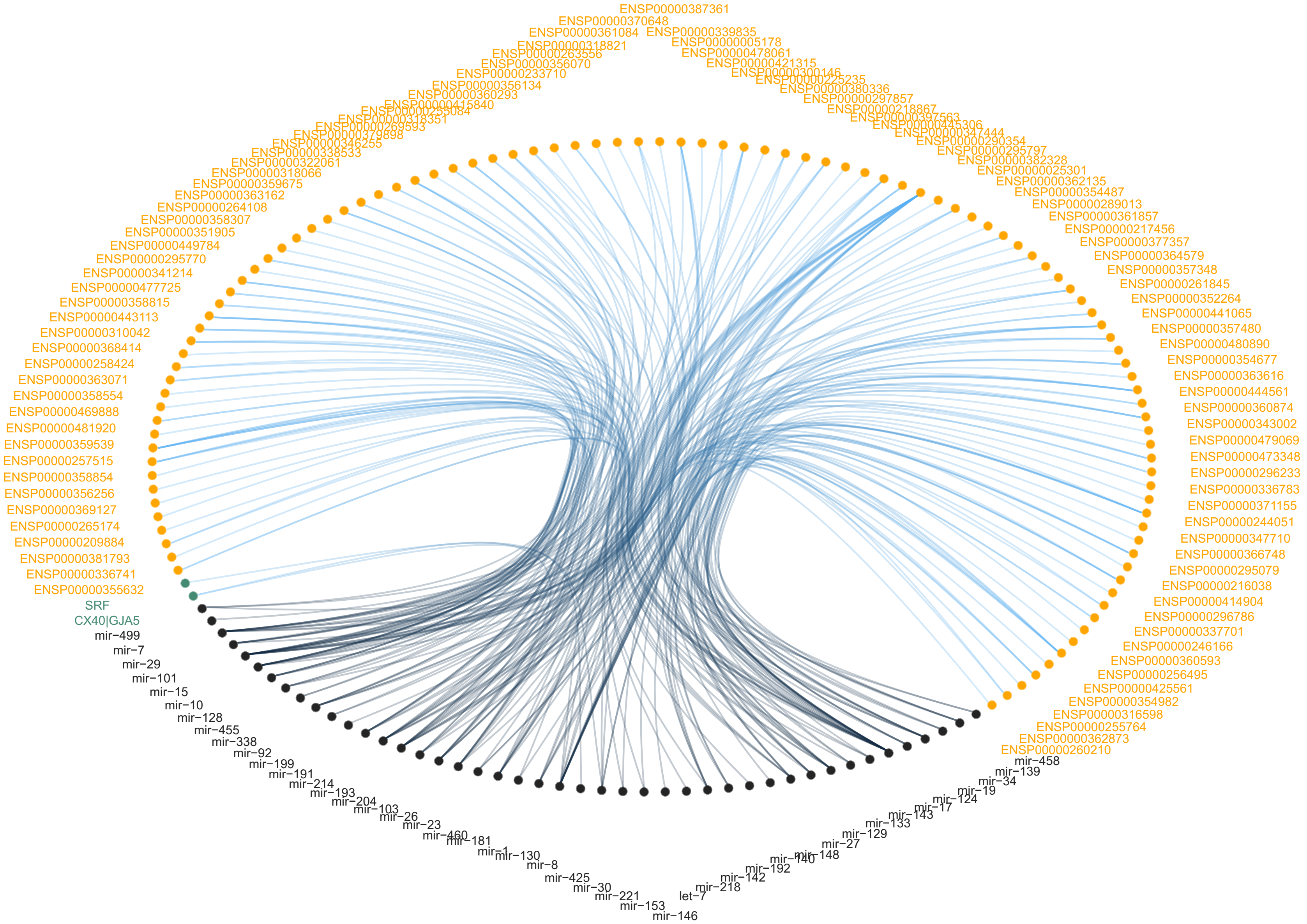

Amphibians X Reptiles

Number of conserved interactions: 232

Supplementary figure S6. Pairwise alignment of miR-target interactions detected in heart of amphibians and reptiles.

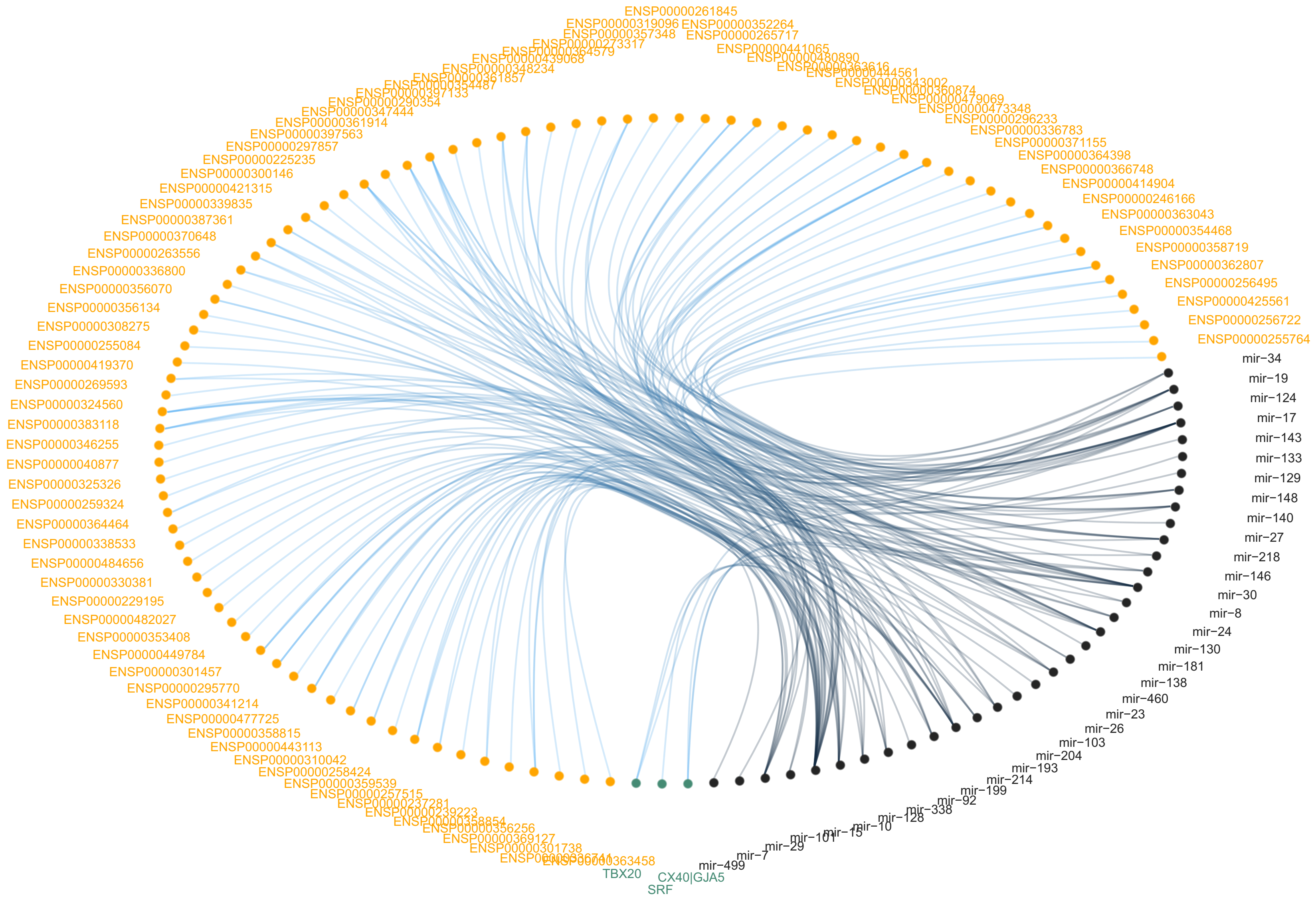

Amphibians X Birds

Number of conserved interactions: 156

Supplementary figure S7. Pairwise alignment of miR-target interactions detected in heart of amphibians and birds.

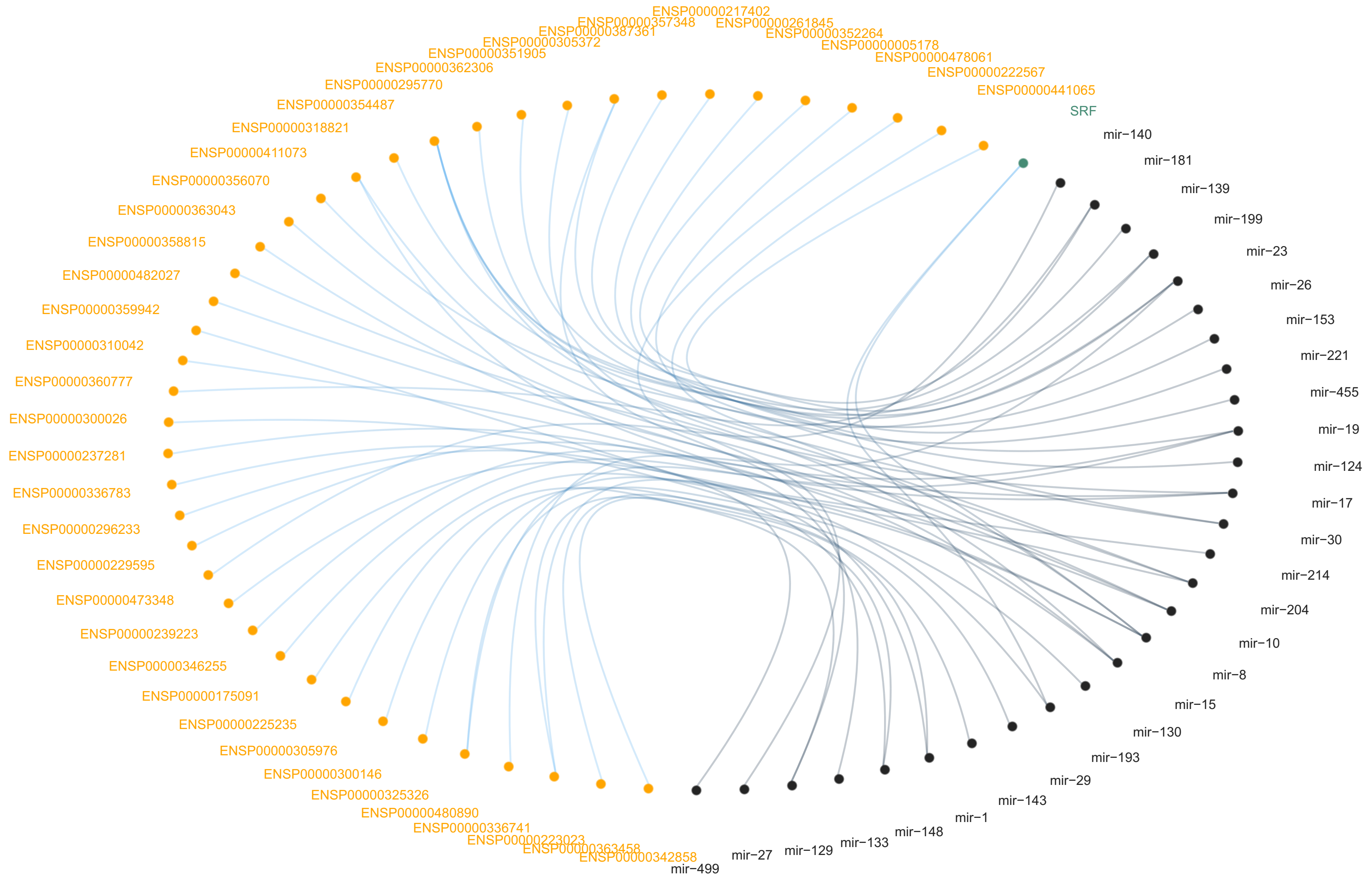

Amphibians X Mammals

Number of conserved interactions: 50

Supplementary figure S8. Pairwise alignment of miR-target interactions detected in heart of amphibians and mammals.

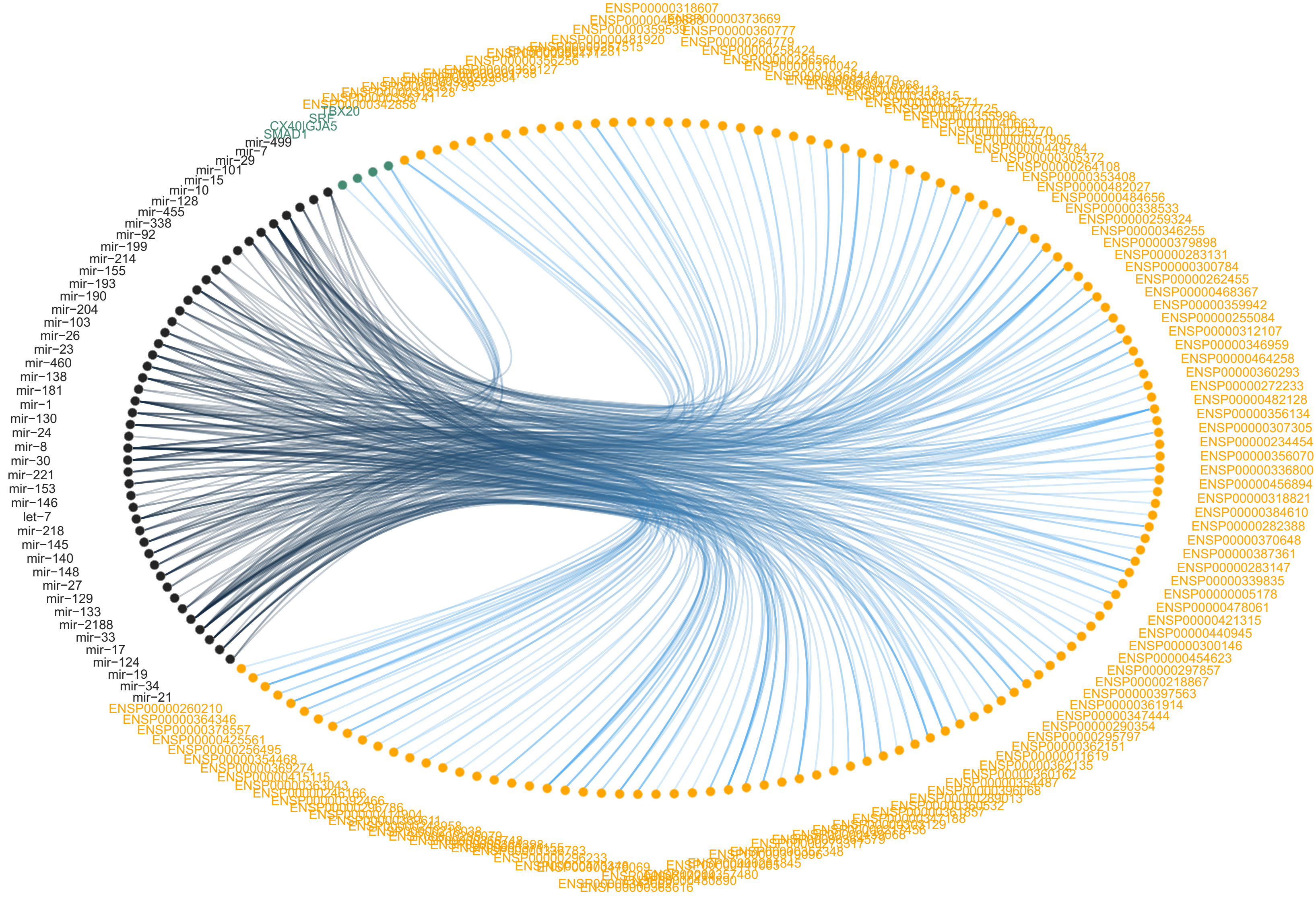

Reptiles X Birds

Number of conserved interactions: 338

Supplementary figure S9. Pairwise alignment of miR-target interactions detected in heart of reptiles and birds.

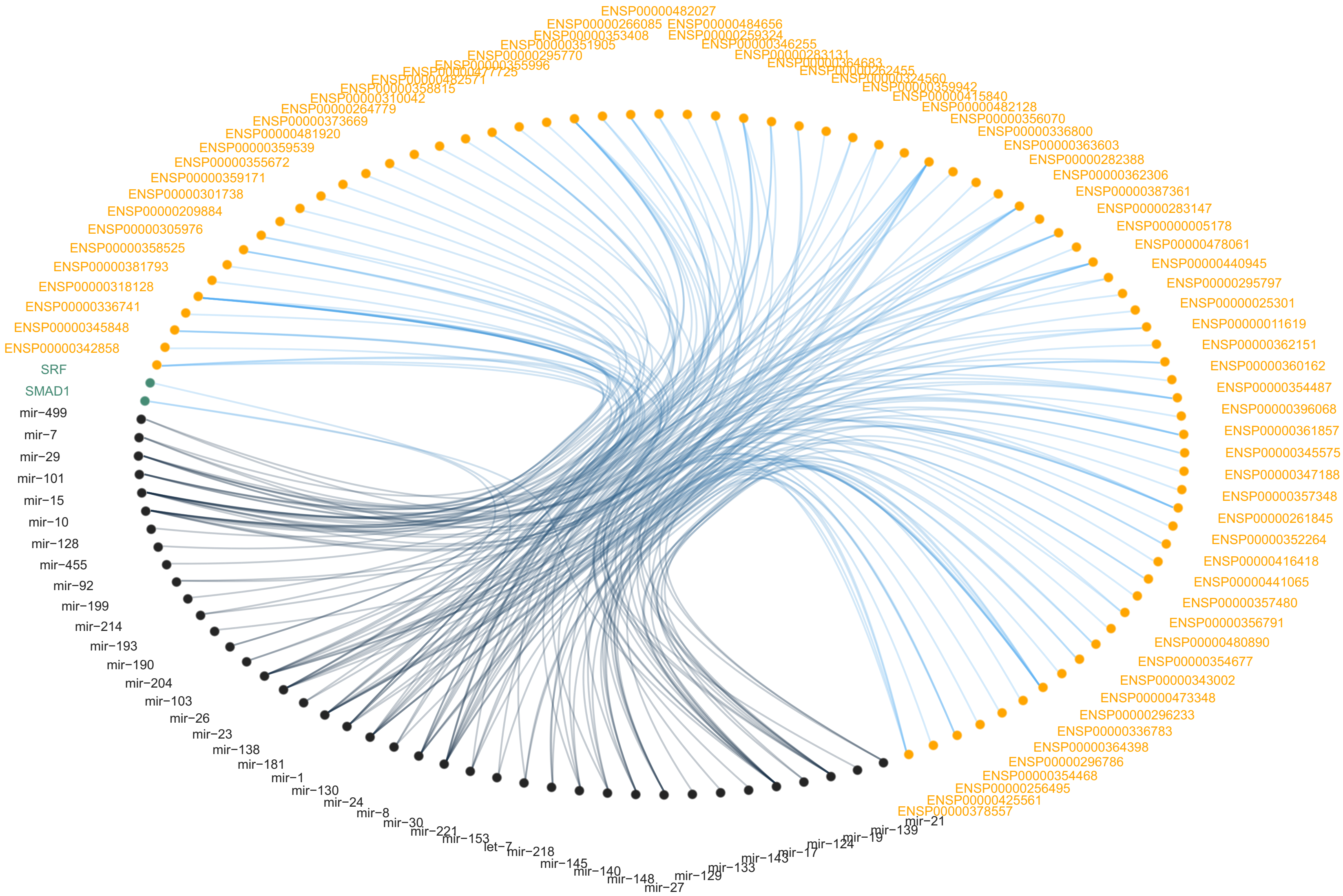

Reptiles X Mammals

Number of conserved interactions: 172

Supplementary figure S10. Pairwise alignment of miR-target interactions detected in heart of reptiles and mammals.

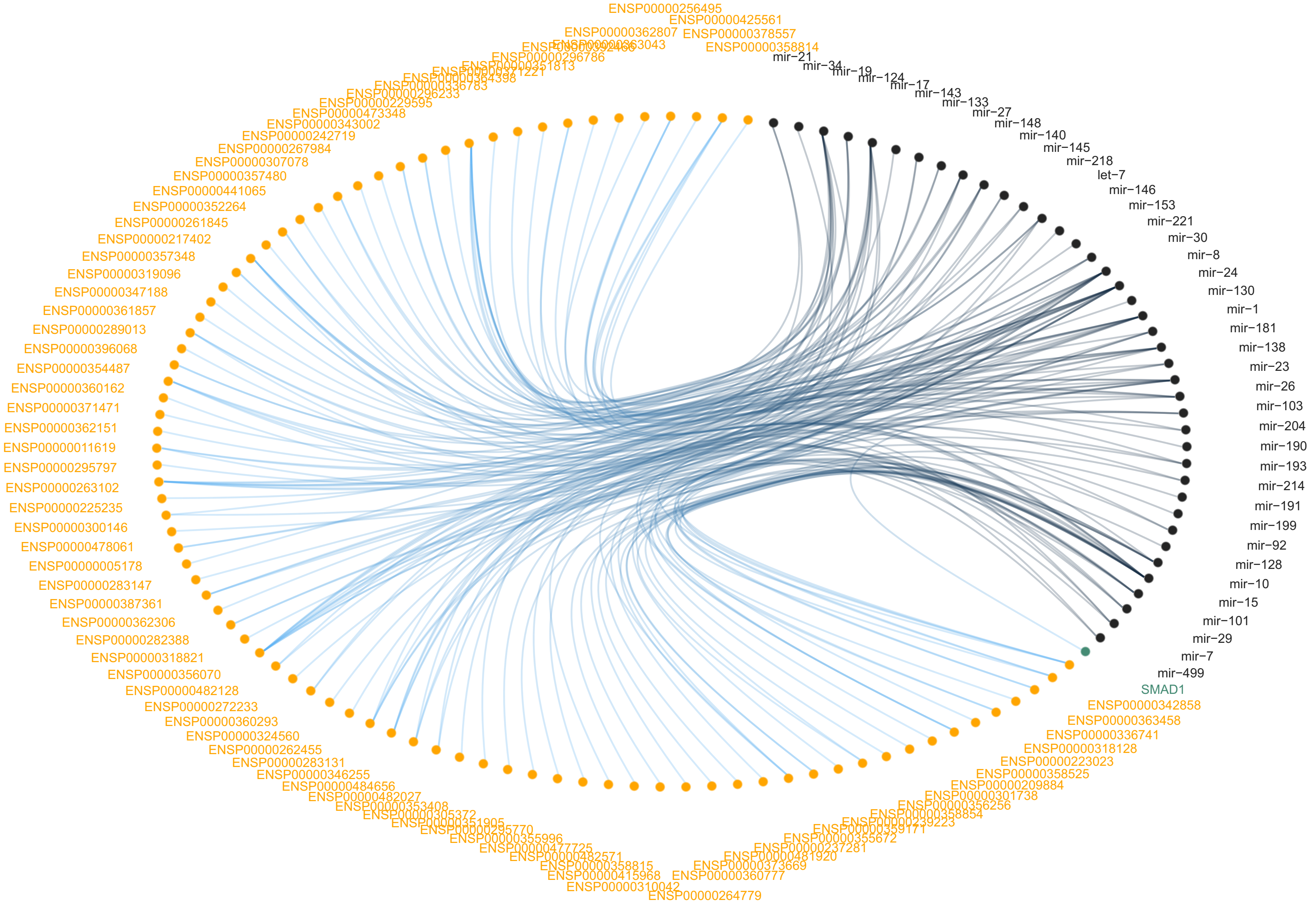

Birds X Mammals

Number of conserved interactions: 159

Supplementary figure S11. Pairwise alignment of miR-target interactions detected in heart of birds and mammals.
