# Supplementary material for: A comparative analysis of heart microRNAs in vertebrates brings novel insights into the evolution of genetic regulatory networks
Source: BMC Genomics. 2021 Mar 4;22:153. doi: 10.1186/s12864-021-07441-4 (PMC7931589; doi:10.1186/s12864-021-07441-4)
Supplement: Supplementary file 7 — Additional file 7 Supplementary data in pdf format. [file 12864_2021_7441_MOESM7_ESM.pdf]

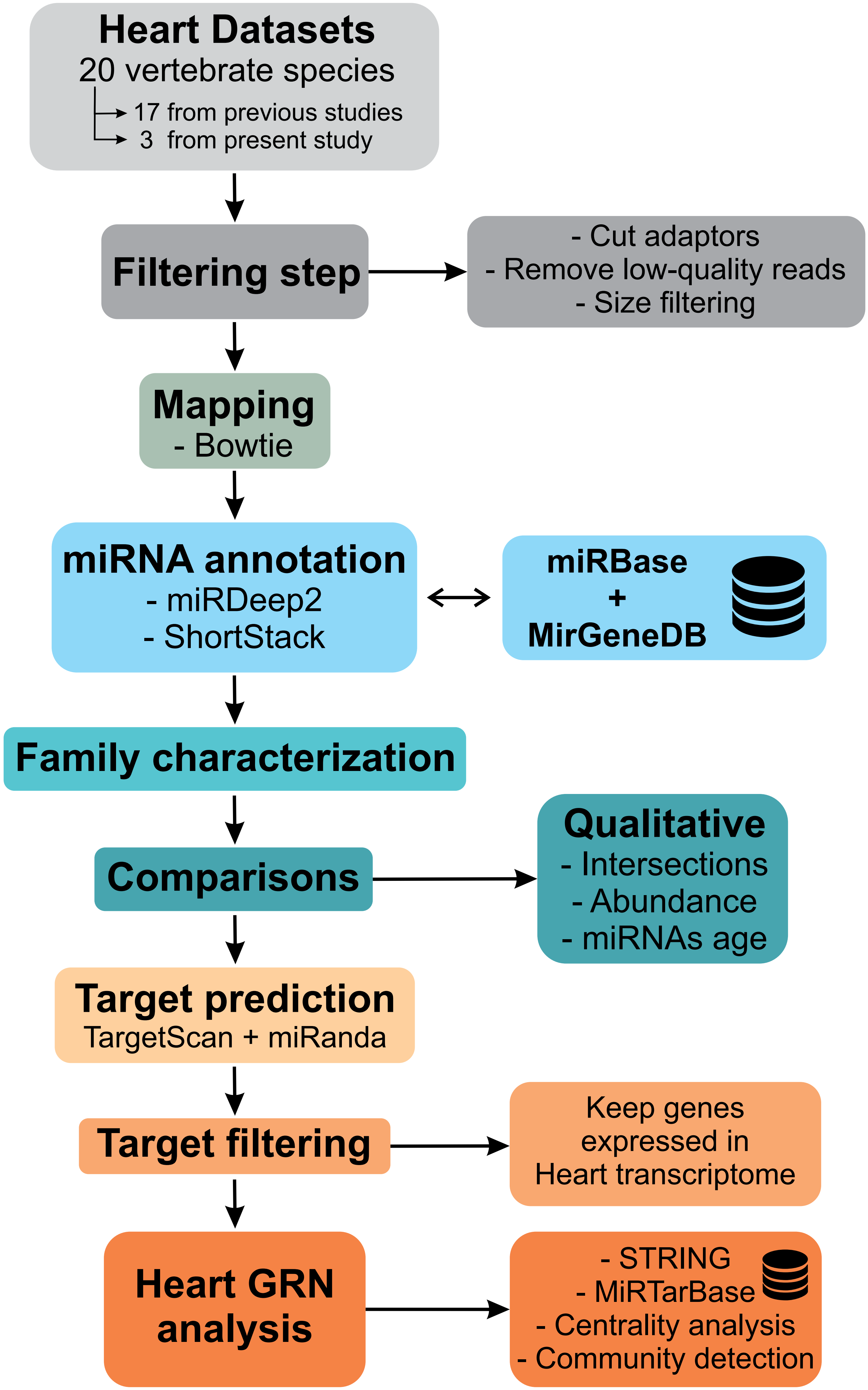
Heart Datasets
20 vertebrate species
17 from previous studies
3 from present study
Filtering step
- Cut adaptors
- Remove low-quality reads
- Size filtering
Mapping
- Bowtie
miRNA annotation
- miRDeep2
- ShortStack
miRBase
+
MirGeneDB
Family characterization
Comparisons
Qualitative
- Intersections
- Abundance
- miRNAs age
Target prediction
TargetScan + miRanda
Target filtering
Keep genes
expressed in
Heart transcriptome
Heart GRN
analysis
- STRING
- MiRTarBase
- Centrality analysis
- Community detection
